# Longitudinal analysis of DNA methylation associated with birth weight and gestational age

**DOI:** 10.1093/hmg/ddv119

**Published:** 2015-04-13

**Authors:** Andrew J. Simpkin, Matthew Suderman, Tom R. Gaunt, Oliver Lyttleton, Wendy L. McArdle, Susan M. Ring, Kate Tilling, George Davey Smith, Caroline L. Relton

**Affiliations:** 1 MRC Integrative Epidemiology Unit, School of Social and Community Medicine, University of Bristol,; 2 School of Social and Community Medicine, University of Bristol, Bristol BS8 2BN, UK and; 3 Institute of Genetic Medicine, Newcastle University, Newcastle upon TyneNE1 3BZ, UK

## Abstract

Gestational age (GA) and birth weight have been implicated in the determination of long-term health. It has been hypothesized that changes in DNA methylation may mediate these long-term effects. We obtained DNA methylation profiles from cord blood and peripheral blood at ages 7 and 17 in the same children from the Avon Longitudinal Study of Parents and Children. Repeated-measures data were used to investigate changes in birth-related methylation during childhood and adolescence. Ten developmental phenotypes (e.g. height) were analysed to identify possible mediation of health effects by DNA methylation. In cord blood, methylation at 224 CpG sites was found to be associated with GA and 23 CpG sites with birth weight. Methylation changed in the majority of these sites over time, but neither birth characteristic was strongly associated with methylation at age 7 or 17 (using a conservative correction for multiple testing of *P* < 1.03 × 10^–7^), suggesting resolution of differential methylation by early childhood. Associations were observed between birth weight-associated CpG sites and phenotypic characteristics in childhood. One strong association involved birth weight, methylation of a CpG site proximal to the *NFIX* locus and bone mineral density at age 17. Analysis of serial methylation from birth to adolescence provided evidence for a lack of persistence of methylation differences beyond early childhood. Sites associated with birth weight were linked to developmental genes and have methylation levels which are associated with developmental phenotypes. Replication and interrogation of causal relationships are needed to substantiate whether methylation differences at birth influence the association between birth weight and development.

## Introduction

Gestational age (GA) and birth weight have been implicated in the determination of long-term health (1–4) and may mediate their persistent biological effects via epigenetic mechanisms (5–8). This is predicated on the notion that epigenetic changes induced *in utero* or early postnatal life persists across the life course; a hypothesis that pervades the Developmental Origins of Health and Disease (DOHaD) literature (9,10) and has been underpinned by observations such as the widely cited Dutch Hunger Winter Studies (11–13).

Previous studies have reported an association between DNA methylation (a form of epigenetic modification) measured in cord blood and GA at delivery (5–7) and birth weight (8). These studies have included either appraisal of preterm birth or GA across the normal range, although it is clear that both have potentially very different determinants and underlying mechanisms.

Previous investigation of the relationship between DNA methylation and GA includes a study by Schroeder *et al*. (5) that detected 41 cytosine bases (CpG sites) showing differential methylation in relation to GA in 39 genes using a cohort of 259 neonates from mothers with a history of neuropsychiatric disorders. They replicated their findings for 26 of these sites (in 25 genes) using a sample of 194 newborns from healthy mothers. Many of the identified CpG sites were within genes implicated in labour, delivery and development of later adverse health outcomes. Similarly, Lee *et al*. (6) identified several differentially methylated regions of the epigenome which were associated with GA. These regions were adjacent to three genes involved in early development (*NFIX*, *RAPGEF2* and *MSRB3*). Further support for an association of DNA methylation and GA comes from a study by Parets *et al*. (7) who performed an epigenome-wide association study (EWAS) on 50 newborns, of whom 22 were preterm deliveries. They identified 9637 CpG sites with methylation levels associated with GA. With respect to birth weight, in a study of 1046 infants, Engel *et al*. (8) reported 19 CpG sites to be associated with cord blood DNA methylation levels; some of the identified CpG sites were within genes that had previously been associated with roles in adipogenesis [*ARID5B* (14)] and DNA repair [*XRCC3* (15,16)]. Tan *et al*. (17) found no epigenome-wide associations between birth weight and methylation from a study of 150 pairs of adult monozygotic twins (median age 57, range 30–74) who were discordant for birth weight. However, they identified three CpG sites in a sub-sample of twins who were extremely discordant for birth weight.

Studies have also been conducted in the context of preterm birth, which by definition is a category of low GA and thus closely correlates with low birth weight (18,19). A recent study (19) identified 1555 CpG sites with differential methylation between term and preterm newborns in a matched case–control study. A key finding was that many of these differences had been resolved by adulthood, suggesting that methylation difference at delivery (when not at term) merely reflects methylation changes during a normal developmental trajectory. Whether these differences in early life affect later phenotypic development has not yet been explored.

Physiological or molecular changes induced in early life have the potential to have profound developmental consequences and implications for health across the life course (20–22). Although previous studies have attempted to consider the persistence of DNA methylation from birth to adulthood, they have been severely limited by analysing DNA methylation at a single time point—either sampled at birth and compared cross-sectionally with birth weight (8) and GA (5–7) or sampled in childhood or adulthood and compared retrospectively with birth weight and GA [or other exposures such as prenatal famine (11)]. ‘Change’ in DNA methylation, i.e. difference detected over time, has been the focus of several studies to date (23–32). Many of these report on cross-sectional samples (26,28), adult populations (25) or both (27,29,30,32). Appraisal of change over time in individuals requires measurement of DNA methylation at more than a single time point. A small number of studies have included two time points of serially measured DNA methylation (23,24,31,33,34). However, while two serial measurements are useful, they are limited to the study of linear change, i.e. the difference in methylation from one clinic to the next.

This study sought to analyse DNA methylation in serial samples from the same individuals at three time points from birth to age 17. Focus was placed on the identification of methylation variable sites associated with birth weight and GA, these being two exposures clearly associated with long-term health consequences. Ten developmental phenotypes (e.g. bone mineral density and weight) were used to identify potential mediation of the birth-development association by DNA methylation.

## Results

There were 914, 973 and 974 samples with blood DNA methylation profiles obtained using the Illumina Infinium HumanMethylation450 BeadChip (Illumina, Inc.) (35). This array measures DNA methylation simultaneously at over 485 000 CpG sites across the human genome. After a quality control (QC) step (see Materials and Methods), we obtained DNA methylation in cord blood (466 432 CpGs), peripheral blood at age 7 years (471 347 CpGs) and peripheral blood at age 15/17 years (469 902 CpGs), respectively. The Accessible Resource for Integrated Epigenomic Studies (ARIES) cohort is gender-balanced, with 49% males; average birth weight was 3.48 kg and GA 39.6 weeks (Table 1). Average maternal age was 29.2 years; 61% of mothers had never smoked, whereas 11% continued to smoke during pregnancy.

**Table 1.** Characteristics of the ARIES sample

	Mean (SD)	*N* (%)
Birth weight	3484 (488)	
Gestational age at delivery	39.6 (1.5)	
<37 weeks		27 (3)
37–41 weeks		821 (90)
>41 weeks		61 (7)
Sex		
Male		445 (49)
Female		469 (51)
Parity	0.7 (0.8)	
Maternal age	29.2 (4.4)	
Maternal smoking		
Never		545 (61)
Quit		248 (28)
Smoker		101 (11)
Delivery method		
Caesarean		83 (9)
Natural		795 (91)

### Gestational age

#### EWAS

Therewas evidence for an association between GA and cord blood methylation at 224 different probes annotated to 155 genes (Table 2 and Supplementary Material, Table S1), after correcting for cell type composition in blood using the method described by Houseman *et al*. (36). GA had a negative association with methylation at 188 probes and a positive association at 36 probes. There was no strong evidence for any associations between GA and peripheral blood methylation measured at age 7 or 15/17.

**Table 2.** Five replicated probes at which cord blood methylation is associated with GA

Probe	EWAS results						Longitudinal analysis results			
	Associated gene	Chr	Coordinates	*t*-statistic	*P*-value	FDR *P*-value	Estimated cord blood methylation (%)	Average yearly change between 0 and 7 (%)	Average yearly change between 7 and 17 (%)	Average difference in childhood yearly change, per week increase in GA at delivery (%)
Probes with a negative association between GA and methylation in the ARIES, WMHP, CANDLE and NB cohorts (5,7)										
cg25551168	*AVP*	20	3 013 343	−5.71	1.70E−08	5.30E−05	54.2	−2.7	n/a	0.10
cg21842274	*CRHBP*	5	76 284 393	−5.51	5.10E−08	1.34E−04	64.3	−1.5	−0.28	0.17
Probes with a negative association between gestational age and methylation in the ARIES, WMHP and CANDLE cohorts (5)										
cg16536918	*AVP*	20	3 013 403	−5.75	1.40E−08	4.60E−05	68.3	−2.9	n/a	0.13
cg01143454	*C20orf141;LOC100288797*	20	2 743 601	−5.43	7.80E−08	1.78E−04	50.9	−3.5	−0.16	0.13
Probes with a positive association between GA and methylation in the ARIES, WMHP, CANDLE and NB cohort (5,7)										
cg05294455	*MYL4*	17	42 641 608	5.71	1.60E−08	5.20E−05	43.2	3.1	0.19	−0.15

#### Replication

Of the 224 probes identified here, 129 were among the 9637 previously reported by the Nashville Birth (NB) cohort (7) [*P* = 1.8e−156, Fisher's exact test (FET)], and 5 were among the 40 reported for Women's Mental Health Program (WMHP) and replicated in Conditions Affecting Neurocognitive Development and Learning in Early Childhood (CANDLE; *P* = 1.3e−11, FET) (5). In each case of common findings, the direction of association is replicated in addition to strong evidence for association. Finally, 72 of the probes (Supplementary Material, Table S1) associated with GA in cord blood overlapped with the 1555 found in an epigenome-wide case–control study of preterm birth (19). Among ARIES, WMHP, CANDLE and NB, there were three probes commonly associated with GA: cg21842274 (*CRHBP)*, cg25551168 (*AVP)* and cg05294455 (*MYL4*) (5,7). Of these, only cg05294455 (*MYL4*) (5,7) was also common to the case–control study of preterm birth (19).

#### Longitudinal analysis

**Figure 1.**
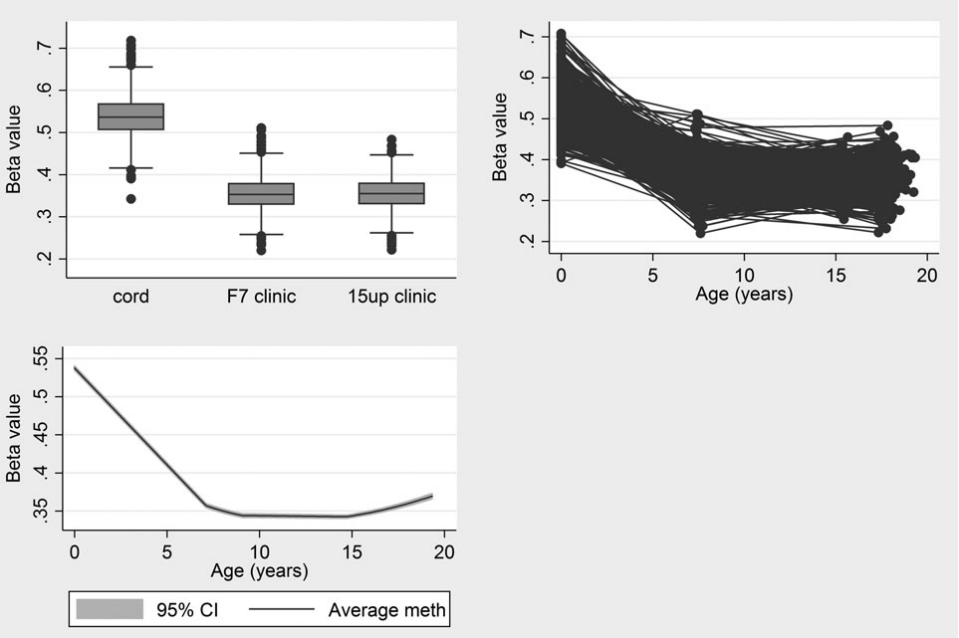
Change in methylation at cg25551168 during childhood and adolescence.

The results of longitudinal analysis of methylation from these 224 probes during childhood and adolescence are provided in Table 2 and Supplementary Material, Table S1. Methylation at the vast majority of the 188 probes showing a negative relationship with GA continued to decrease during childhood. For example, the change in methylation at cg25551168 (*AVP*) is shown in Figure 1. From an estimated 54.2% in cord blood, methylation decreased by 2.7% per year on average during early childhood and then reached a plateau during adolescence with negligible change in methylation from 7 to 17 years. This pattern of rapid change during early childhood followed by stabilization during adolescence is evident across the majority of CpG sites.

Of the 188 negatively associated probes, there were 160 where GA was associated with childhood changes (from 0 to 7 years) in methylation (i.e. where an interaction between GA and age was found). Almost all of the observed interactions are positive, which suggests that those children with a shorter gestation period have faster methylation change during childhood, i.e. methylation differences at birth are resolving during childhood. There was no strong evidence for an effect of GA on methylation changes from age 7 to 17.

Of the 36 probes which had increased methylation per week of gestation, 34 continued to increase in methylation during childhood. The maximum increase was 6.7% per year between birth and 7 years (*EBF4*). However, just 5 of the 36 had an increase in methylation between 7 and 17 years, suggesting that methylation levels had largely stabilized by age 7. An interaction between GA and methylation changes from birth to age 7 was identified for 31 of the 36 positively associated probes. Each of these suggests that children with shorter gestation have a faster rate of change in methylation during childhood, which again suggests that methylation differences attributed to GA are resolving during early life. For example, the average cord blood methylation at cg25551168 (*AVP*) was 54.7% and for each week of GA, methylation was 0.7% lower on average, such that children with GA of 40 weeks would be estimated to have a 7% lower methylation than those with GA of 30 weeks, on average. We found that methylation decreased by 2.7% per year but that for each extra week of GA the change in methylation is slowed down by 0.1% per year. Thus, children with a 40-week GA at delivery would lose 1.7% methylation per year, compared with 2.7% per year for children with 30-week GA, on average. Thus, at age 7, children with GA of 30 or 40 weeks would have equal methylation on average, explaining a lack of an association between GA and methylation or methylation change at or beyond age 7.

### Birth weight

#### EWAS

We identified 23 probes in 14 genes where cord blood methylation was associated with birth weight (7 probes did not have a RefSeq gene annotation; Table 3). Birth weight was positively associated with cord blood methylation at 10 probes and negatively associated at 13. There was no strong evidence for an association between birth weight and peripheral blood methylation at age 7 or 15/17.

**Table 3.** Probes at which cord blood methylation is associated with birth weight

Probe	EWAS results						Longitudinal analysis results			
	Associated gene	Chr	Coordinates	*t*-statistic	*P*-value	FDR *P*-value	Average cord blood methylation (%)	Average yearly change between 0 and 7 (%)	Average yearly change between 7 and 17 (%)	Average difference in childhood yearly change, per kg increase in BWT (%)
Probes with a negative association between birth weight and methylation in both the ARIES and MoBa cohorts (8)										
cg20076442	NA	8	72 907 751	−5.89	6.01E−09	2.92E−04	72.4	−4.3	−0.11	0.38
cg25953130	ARID5B	10	63 423 556	−5.83	8.23E−09	3.31E−04	55.1	n/a	n/a	0.60
Probes with a negative association between birth weight and methylation in the ARIES cohort only										
cg04521626	PLD2	17	4 661 168	−6.60	8.01E−11	3.89E−05	57.2	−3.5	n/a	0.40
cg14097568	NA	1	94 565 530	−6.18	1.06E−09	1.62E−04	53.7	−1.9	−0.51	0.37
cg17133774	CHD5	1	6 121 254	−6.06	2.17E−09	2.11E−04	30.0	−2.8	n/a	0.74
cg00654448	NA	8	142 434 497	−5.93	4.83E−09	2.61E−04	16.5	−0.8	n/a	0.38
cg00442282	RARA	17	35 724 590	−5.84	7.77E−09	3.31E−04	25.3	−1.1	−0.08	0.39
cg13696490	LOC201651	3	152 970 696	−5.82	8.87E−09	3.31E−04	66.6	0.4	−0.47	0.37
cg12044213	CCHCR1;TCF19	6	31 232 957	−5.69	1.83E−08	5.54E−04	67.0	−2.0	−0.27	0.23
cg08817867	NA	17	19 597 146	−5.65	2.29E−08	6.16E−04	42.7	−1.9	n/a	0.62
cg00382138	CFI;CFI	4	110 942 748	−5.64	2.50E−08	6.39E−04	67.6	−1.1	−0.17	0.56
cg06870470	DOCK6	19	11 176 767	−5.58	3.39E−08	8.24E−04	34.3	−3.3	n/a	0.29
cg25557739	NA	14	96 814 038	−5.46	6.73E−08	1.49E−03	41.5	−1.2	−0.24	0.09
Probes with a positive association between birth weight and methylation in the ARIES cohort only										
cg24324628	NHSL1	6	138 908 575	6.41	2.62E−10	6.36E−05	80.8	0.9	−0.06	−0.28
cg15783941	NFIX	19	12 993 221	6.14	1.33E−09	1.62E−04	75.4	1.4	n/a	−0.35
cg14597739	LTA	6	31 647 977	6.03	2.63E−09	2.13E−04	65.3	0.2	0.11	−0.39
cg22962123	HOXA3	7	27 120 130	6.00	3.12E−09	2.17E−04	81.2	1.1	n/a	−0.38
cg05851442	HOXA3	7	27 119 737	5.94	4.55E−09	2.61E−04	79.8	1.3	n/a	−0.46
cg23387597	ITPRIP	10	106 083 768	5.77	1.15E−08	3.98E−04	82.9	0.4	0.05	−0.30
cg24973755	MAEA	4	1 294 972	5.70	1.75E−08	5.54E−04	76.3	0.2	−0.15	−0.42
cg16219283	LTA	6	31 647 981	5.68	1.94E−08	5.54E−04	68.0	n/a	0.13	−0.27
cg25799241	NA	8	144 877 728	5.46	6.46E−08	1.49E−03	68.2	1.9	n/a	−0.32
cg06658067	NA	16	29 301 844	5.45	7.05E−08	1.49E−03	73.6	n/a	−0.04	−0.17

#### Replication

Of the 23 associations observed between birth weight and cord blood methylation, two had been previously shown to have a negative association (8) in the Norwegian Mother and Child (MoBa) cohort (37). These were cg20076442 (no RefSeq gene) without a local gene, and cg25953130 (*ARID5B*).

#### Longitudinal analysis

**Figure 2.**
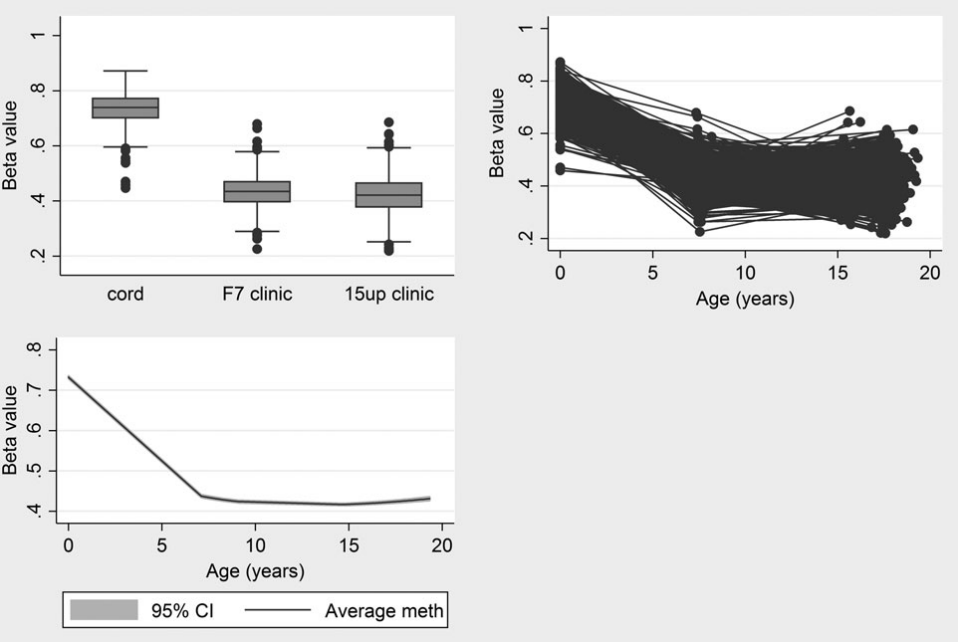
Change in methylation at cg20076442 during childhood and adolescence.

Longitudinal analysis of methylation at these probes showed that 11 of the 13 sites negatively associated with birth weight had a reduction in average methylation during childhood (Table 3). Beyond age 7, 7 of these 13 probes demonstrated a continued reduction in average methylation levels. The most pronounced change in methylation occurred in cg20076442 (no RefSeq gene). Cord blood methylation was estimated to be 72.4% and this decreased by 4.3% per year from birth to age 7 on average. This reduction in methylation continued throughout later childhood and adolescence, but at a rate of 0.11% per year (Fig. 2).

Probes with methylation levels which were positively associated with birth weight tended to increase in methylation during childhood and adolescence. During childhood (age 0–7), an interaction between birth weight and age was found for each of the 23 probes. Across all probes, lower birth weight was associated with faster changes in methylation during childhood, i.e. birth weight-related methylation differences resolved during childhood. There was no strong evidence that birth weight was associated with methylation change between ages 7 and 17.

### Exploration of phenotypic differences in adolescence

With 224 identified probes in the EWAS of GA and 10 selected developmental phenotypes (height, weight, leg length, lean mass, fat mass, bone mass, bone mineral density, IQ, forced expiratory volume and forced vital capacity), there were a possible 2240 association between GA-related methylation and developmental phenotypes. Of these, 70 probe–phenotype associations were observed, yet just two GA–phenotype associations were observed (with leg length and IQ). However, neither of these associations overlapped so there is no support in this data set for methylation differences in these probes being associated with later phenotypic differences.

**Figure 3.**
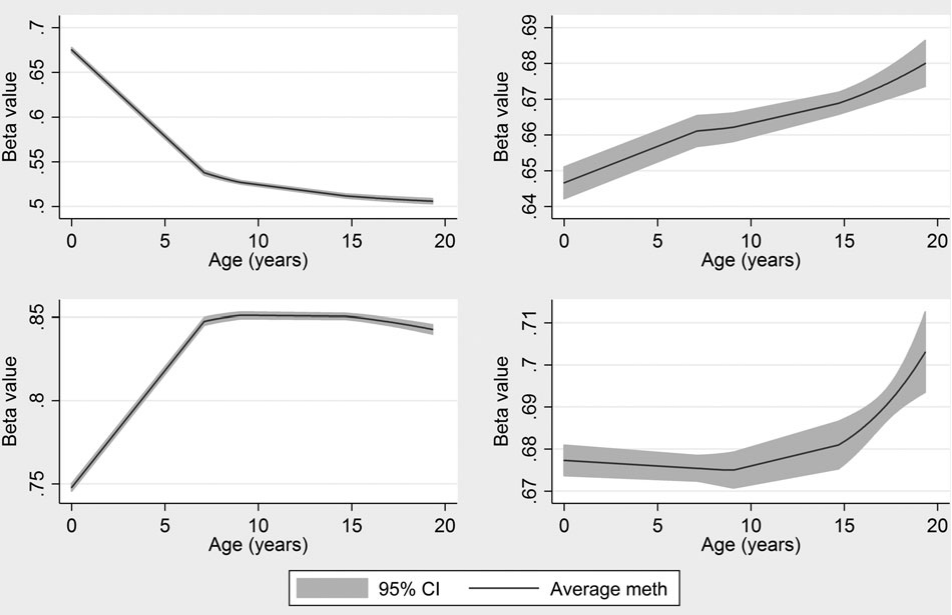
Change in methylation at those sites found to be associated with birth weight and later developmental phenotypes.

In the birth weight EWAS, 23 probes were identified such that 230 probe–phenotype association tests were performed. Of these, 14 probe–phenotype associations were observed while all 10 developmental phenotypes were associated with birth weight. The 14 overlapping associations are presented in Table 4. There was a strong positive association between birth weight and development and a negative relationship between methylation and development in 12 of the 14 phenotypes (i.e. lower methylation was associated with better development). Of the 14 associations, eight involved methylation at cg15783941(*NFIX*) and five were found in two CpG sites in the *LTA* gene. Longitudinal analysis showed that estimated cord blood methylation at cg15783941 (*NFIX*) is 75.4% and this increases by 1.4% per year on average during childhood with no evidence for further methylation change beyond age 7 (Fig. 3). Given the observed association between cord blood methylation and later phenotypes, there is support here for differences at birth having longer term effects.

**Table 4.** Results of developmental phenotypes' association with DNA methylation and birth weight

Phenotype	Probe	Gene	*t*-statistic (methylation)	*P*-value (methylation)	*t*-statistic (birth weight)	*P*-value (birth weight)
Lean mass 17	cg12044213	*CCHCR1;TCF19*	4.32	1.76E−05	14.95	<1E−16
Height 17	cg14597739	*LTA*	−4.24	2.49E−05	18.13	<1E−16
Lean mass 17	cg14597739	*LTA*	−4.75	2.42E−06	14.95	<1E−16
FVC 15	cg14597739	*LTA*	−4.06	5.37E−05	11.82	<1E−16
FVC 15	cg15783941	*NFIX*	−6.71	3.87E−11	11.82	<1E−16
Weight 17	cg15783941	*NFIX*	−4.65	3.91E−06	15.59	<1E−16
Bone mass 17	cg15783941	*NFIX*	−6.73	3.21E−11	16.31	<1E−16
Fat mass 17	cg15783941	*NFIX*	4.14	3.91E−05	5.26	1.42E−07
BMD 17	cg15783941	*NFIX*	−5.55	3.97E−08	8.74	<1E−16
Height 17	cg15783941	*NFIX*	−9.05	1.07E−18	18.13	<1E−16
FEV1 15	cg15783941	*NFIX*	−5.60	2.96E−08	12.15	<1E−16
Lean mass 17	cg15783941	*NFIX*	−10.96	4.56E−26	14.95	<1E−16
FVC 15	cg16219283	*LTA*	−3.85	1.28E−04	11.82	<1E−16
Lean mass 17	cg16219283	*LTA*	−3.72	2.10E−04	14.95	<1E−16

## Discussion

### Summary

We identified 224 CpG sites where cord blood methylation was found to be associated with GA across the normal range of gestation. These included an appreciable overlap with previously identified associations including three probes that have previously been reported in three separate cohorts (5,7) and one probe which was common to all four cohorts (5,7,19). An EWAS of birth weight discovered associations with DNA methylation at 23 CpG sites. Of these, two probes have been previously identified in the MoBa cohort (8). There was little evidence of an association between methylation at age 7 or 17 years and either of birth weight or GA. This is suggestive of the non-persistence of birth-related DNA methylation differences and implies that much of the variation in birth-related DNA methylation observed at birth attenuates markedly in the first few years of life. Indeed, there appears to be a phase of rapid ‘catch-up’ in methylation differences. This observation has important implications for the role of epigenetic processes in developmental programming (in blood); although it does not preclude the involvement of epigenetic mechanisms *per se*, it suggests that any downstream consequences of these specific epigenetic perturbations observed at birth may be set in train during early life rather than the marks themselves persisting across the life course. These inferences are limited to differential methylation associated with GA and birth weight and may not extend to all changes induced *in utero*, exemplified by the smoking-responsive changes in DNA methylation recently reported which can persist for longer periods of time (38,39).

Longitudinal analysis provided evidence that methylation levels change rapidly during early development. For the majority of probes with changing methylation levels, this change was more pronounced during the immediate postnatal years, with many sites tending to stabilize in methylation level beyond age 7. Where interactions were found, lower birth weight and shorter GA were associated with an increased rate of methylation change during the life course, suggesting a ‘catch-up’ mechanism in early life. The resolution of cord blood methylation differences during childhood has been recently reported (40) at differentially methylated probes (DMPs) related to pre-pregnancy maternal BMI. However, this is in contrast to changes in methylation at smoking-responsive probes, where differences in cord blood methylation associated with maternal smoking have been shown to persist through childhood and adolescence (39). Two previous studies have sought to investigate methylation changes in early life, from birth to 6 months (34) and from birth to 18 months in twins (33). Using two time points, they performed paired *t*-tests and established that 30% of 330 168 probes under investigation had methylation which changed in the first 18 months, with an average increase of 3.1% per year (33). In contrast, our longitudinal analysis was not epigenome-wide and investigated change in only those DMPs identified in cord blood in relation to birth weight or GA. The questions addressed are therefore different; one establishing the difference in methylation across the methylome between two ages, and the other establishing the extent of change over time in loci shown to be differentially methylated with regard to a specific trait. In contrast to the 1% of the probes previously shown to differ between birth and 12 months of age (5), our study suggests that the vast majority of GA- (87%, 190/224) and birth weight (87% 20/23)-associated DMPs undergo a period of rapid change. The lower estimate of change found by Martino *et al*. (34) may relate to the cell type studied, CD4+ cells rather than whole blood cells and the shorter time period, 1 year compared with the 7 years in the current study. Moreover, our findings do not necessarily contradict the previous results because the CpG sites perturbed by GA and birth weight are likely to be specifically targeted by the ‘catch-up’ mechanism discussed above that resolves GA and birth weight-related changes in early life.

Birth weight-related cord blood methylation in the *NFIX* gene was shown to be associated with developmental phenotypes, which were also associated with birth weight, including bone mineral density at 17 years of age. *NFIX* regulates the development of the brain and of bone and skeletal muscle. *NFIX* has been identified previously as having birth weight-associated levels of DNA methylation (6). Birth weight-associated sites near the transcription start of *LTA* were also associated with developmental phenotypes including lean mass. *LTA* is a tumour necrosis factor family member produced by lymphocytes and is integrally involved in the development of secondary lymphoid organs both before and shortly after birth (41) as well as in a wide variety of immune responses. Previous work has also identified likely associations between cord blood methylation in the region of the endothelial nitric oxide synthase gene (*eNOS*) and bone mineral density at 9 years (42). We observed an association with GA in a neighbouring gene promoter (*KCNH2*) less than 40 Kb away.

### Comparison with previous EWAS

Our analyses replicate the findings of several previous studies (5,7,8). Among these are two GA-related probes in the *AVP* gene, which has been linked together with the *OXT* gene to the timing of delivery (43). Suggestively, nearby associated probes were identified in the promoter of *OXT* in our cohort ARIES and in WMHP and CANDLE (5) though not at precisely the same probes. Another is a probe adjacent to the transcription start site of the *CRHBP* gene. *CRHBP* encodes a protein produced by the placenta, but that drops dramatically prior to parturition in order to promote corticotropin-releasing hormone activity (7). Whereas previous studies identified an association near the *ESR1* gene, also known to play a role in parturition, no such associations were found in the ARIES cohort. A third associated probe (cg05294455) is located about 100 bp upstream of the *MYL4* gene that encodes a motor protein involved in muscle contractions. Salomonis *et al.* (44) show that *MYL4* is one of the most down-regulated transcripts in the mouse myometrium during late gestation and note that the *MYL4* plays a primary role in uterine contraction at term. In human cord blood, we show that *MYL4* methylation is positively associated with GA possibly implying a negative association with *MYL4* gene expression in contrast to a positive association in the myometrium. This is consistent with the observation of Cruickshank *et al*. (19) of large methylation decrease at the same probe in neonatal blood spots of preterm infants compared with term infants. The relationship between blood DNA methylation of these parturition-related genes and timing of delivery remains to be elucidated.

Of the three regional associations identified by Lee *et al*. (6), we replicated one near the *MSRB3* gene encoding a product that catalyzes the reduction of methionine sulphoxide to methionine. It has been hypothesized that increased levels of methionine sulphoxide in body tissues contribute to aging (45,46). The negative association between DNA methylation and GA that we observe suggests that *MSRB3* may be more highly expressed in cord blood at later GAs.

Unlike Paret *et al.* (7), we did not observe associations at probes near key epigenetic genes *DNMT1*, *DNMT3A*, *DNMT3B* and *TET1*. However, we did observe associations at the same sites near the *CHD4* and *CHD5* genes that encode members of a nucleosome remodelling and deacetylase complex. Paret *et al.* (7) observe positive associations at probes near *MMP9*, which is involved in the breakdown of the extracellular matrix in the process of cervical ripening (47). We similarly observe positive associations at probes near *MMP15*, another matrix metalloproteinase involved in extracellular matrix breakdown.

Two of the 23 birth weight-related probes have previously been identified in the MoBa cohort (8), including the same probe in *ARID5B*. This gene has been shown to be associated with postnatal adiposity in mice (14). As noted for the MoBa cohort (8), several birth weight-related sites (6) are linked to genes that play important roles in development, including *RAR*, *NFIX*, *LTA* and *HOXA3*. For example, knockout of *RAR* in mice induces lethality shortly after birth and results in testis degeneration (48), *NFIX* provides foetal-specific transcription regulation in developing skeletal muscle (49), *LTA* is critically involved in the development of secondary lymphoid organs both before and shortly after birth (50), and *HOXA3* is involved in patterning the cranial neural crest (51). There was no overlap between our birth weight-related probes and three probes found in a study of extremely birth weight discordant adult twins (17).

### Strengths

A major strength of our analysis is the ARIES data set, which contains serially measured DNA methylation on 1018 children along with a plethora of phenotypic information. This has enabled longitudinal analysis of DNA methylation over three measurement occasions. Using these data, we have provided an in-depth study of methylation changes during childhood and adolescence at methylation loci associated with birth weight and GA.

### Limitations

Although we corrected for cellular heterogeneity using the Houseman (36) algorithm, there is a different ratio of white blood cell types in cord blood compared with peripheral blood drawn in childhood. This raises the possibility that differences observed can be explained by longitudinal (possibly developmental) changes in white blood cell profiles. However, we used independent surrogate variable analysis (ISVA) estimated components to attempt to correct for changing blood composition. Due to the unique nature of our data set and the lack of available serial data in other cohort studies (at the current time), we have not been able to replicate our longitudinal analysis findings in an independent cohort, although our findings do show concordance with other cross-sectional studies. Modelling methylation over time at single sites rather than over regions has inherent limitations. Furthermore, with only three repeated measures of methylation available, our longitudinal modelling strategy was limited. For example, with several repeated measures available, smoothing methods (52) may have been employed to accurately capture the pattern of methylation change. With respect to interpretation, more detailed modelling of correlated groups of probes or differentially methylated regions of the genome would help to enhance the functional relevance of our observations. Another limitation is tissue specificity. Although convenient, use of blood is not likely the most relevant tissue for investigating the effects of birth characteristics on developmental phenotypes. It is possible that DNA methylation perturbations in tissues that actually play a role in the phenotypes (e.g. brain tissues and IQ) might actually persist throughout childhood. The limited association observed between DNA methylation at birth and later phenotype may also be related to the stringent cut-offs imposed in our study and consideration of a larger pool of DMPs may uncover more evidence of phenotypic consequences of birth weight- and GA-associated differential methylation.

### Conclusion

Using serially collected DNA methylation, we have provided strong evidence for the non-persistence of epigenetic marks that are associated with birth characteristics throughout childhood and adolescence. In the vast majority of probes identified in our study, methylation levels change rapidly in the early stages of development and then stabilize into adolescence. While birth weight appears to have an influence on methylation changes, there is less evidence that GA plays a role in the evolution of long-term DNA methylation patterns. There is evidence that birth weight-related methylation differences may be linked to later developmental phenotypes. Replication in other tissue types and the application of causal analysis methods are needed to further address this hypothesis, namely that the effect of birth weight on later developmental phenotypes is influenced in part by DNA methylation differences at birth.

## Materials and Methods

### Study population

This study used DNA methylation data generated under the auspices of the Avon Longitudinal Study of Parents and Children (ALSPAC) (53). ALSPAC recruited 14 541 pregnant women with expected delivery dates between April 1991 and December 1992. Of these initial pregnancies, there were 14 062 live births and 13 988 children who were alive at 1 year of age. The study website contains details of all the data that are available through a fully searchable data dictionary (http://www.bris.ac.uk/alspac/researchers/data-access/data-dictionary).

As part of the ARIES project (http://www.ariesepigenomics.org.uk), a sub-sample of 1018 ALSPAC child–mother pairs had DNA methylation obtained using the Infinium HumanMethylation450 BeadChip (Illumina, Inc.) (35). In this study, we use DNA methylation data generated from cord blood and peripheral blood samples at age 7 and again at age 15 or 17 years, leading to three measurements of DNA methylation per child. However, DNA methylation data were also obtained from the mothers of these children, from blood samples taken during pregnancy and at a follow-up clinic 18 years later.

### Laboratory methods, QC and preprocessing

All DNA methylation wet laboratory and preprocessing analyses were performed at the University of Bristol as part of the ARIES project. Following extraction, DNA was bisulphite-converted using the Zymo EZ DNA Methylation™ kit (Zymo, Irvine, CA, USA). Infinium HumanMethylation450 BeadChips were used to measure genome-wide DNA methylation levels at over 485 000 CpG sites. The arrays were scanned using an Illumina iScan, with initial quality review using GenomeStudio. The assay detects methylation of cytosine at CpG islands using two site-specific probes—one to detect the methylated (M) locus and one to detect the unmethylated (U) locus. Single-base extension of the probes incorporates a labelled chain-terminating ddNTP, which is then stained with a fluorescence reagent. The ratio of fluorescent signals from the methylated site versus the unmethylated site determines the level of methylation at the locus. The level of methylation is expressed as a ‘beta’ value (*β*-value), ranging from 0 (no cytosine methylation) to 1 (complete cytosine methylation). *β*-values are reported as percentages.

During the data generation process, a wide range of batch variables were recorded in a purpose-built laboratory information management system (LIMS). The LIMS also reported QC metrics from the standard control probes on the 450 K BeadChip. Samples failing QC (average probe detection *P*-value ≥0.01) were repeated. Samples from all time points in ARIES were randomized across arrays to minimize the potential for batch effects. As an additional QC step, genotype probes on the 450 K BeadChip were compared between samples from the same individual and against SNP-chip data to identify and remove any sample mismatches. In addition to these QC steps, probes that contained <95% of signals detectable above background signal (detection *P*-value <0.01) (*N* = 7938) were excluded from analysis. After excluding these probes, as well as control probes and probes on sex chromosomes, a total of 466 432 CpGs were included in the main analysis for cord blood methylation. At age 7, 471 347 CpGs were included and at age 17, 469 902 CpGs were included in the main analysis, following the same exclusion criteria. Raw *β*-values were preprocessed using R (version 3.0.1) with background correction and subset quantile normalization performed using the pipeline described by Touleimat and Tost (54). *β*-values were corrected for cell type heterogeneity in blood using the method described by Houseman *et al*. (36).

### Statistical analysis

#### EWAS

Using CpGassoc (55), separate EWASs of GA and birth weight with cord blood methylation, peripheral blood methylation at age 7 and peripheral blood methylation at age 15/17 years were carried out. As well as being mutually adjusted for GA and birth weight, each EWAS was adjusted for parity, maternal age, maternal smoking, child sex and delivery method (caesarean yes/no) as potential confounders. DNA methylation *M*-values (i.e. logit-transformed *β*-values) were used in each EWAS (56). We report those CpG sites where an association was found at the *P* < 1.03 × 10^−7^ level (to account for multiple testing across CpG sites). A description of each CpG site, as defined by the Illumina BeadChip probe name and RefSeq annotation (where known), is presented.

#### Replication

Two previous studies have used Illumina BeadChip arrays to identify associations with GA, the first using the 27 K BeadChip in the WMHP cohort and the CANDLE cohort (5), and the second using the 450 K BeadChip in the NB cohort (7). The MoBa cohort (37) has investigated relationship between birth weight and DNA methylation using the 450 K BeadChip (8). We compared identified probes with those found in these EWAS [of GA (5,7) and birth weight (8)], highlighting any replicated CpG sites. We also compared the results of our GA EWAS with findings from a case–control study of preterm birth (19), which used the 450 K BeadChip.

#### Longitudinal analysis

Longitudinal DNA methylation data from birth to 17 years were analysed for those CpG sites found to be associated with the birth weight or GA. Methylation *β*-values from all three time points were normalized together using the method described by Touleimat and Tost (54) and ISVA (57) was used to generate the top 20 components of variation. These components account for confounding due to position effects and also any changing cell type proportions. The methylation *β*-values were modelled over time in the children using multilevel models (58,59) to account for within- and between-child variation in methylation. Multilevel models also allow investigation of both methylation change during childhood and the effect of birth weight/GA on this methylation change. Irregular measurements of methylation over time (i.e. some children measured at 15 and some at 17) are handled naturally by multilevel models, introducing no bias from this design. A linear spline term was added to allow for different linear changes from 0 to 7 and from 7 to 17. For example, the model for GA-related methylation changes is

$$\begin{array}{ll} \text{met}{\text{h}}_{ij} & =\beta_{0}+u_{0i}+\beta_{1}\text{GA}+\beta_{2}\text{ag}{\text{e}}_{ij}+\beta_{3}(\text{ag}{\text{e}}_{ij}-7{)}_{+}\\ & \;\;+\beta_{4}\text{GA}\times \text{ag}{\text{e}}_{ij}+\beta_{5}\text{GA}\times (\text{ag}{\text{e}}_{ij}-7{)}_{+}+\epsilon_{ij} \end{array}$$

$$\epsilon_{ij}∼N(0,\;\sigma_{\epsilon }^{2})$$

$$u_{0i}∼N(0,\sigma_{u}^{2})$$

where i indexes the children in ARIES, j=1,2,3 indexes the measurement occasion, $a_{+}\;=a$ if a>0 or 0 otherwise and $u_{0i}$ is a random intercept, which allows children to have different cord blood methylation. *β*_1_ gives the average change in methylation per week increase in GA at delivery; *β*_2_ gives the average change in methylation from birth to adolescence; *β*_3_ is the change to this trend (i.e. *β*_2_) from 7 to 17; *β*_4_ is the difference in methylation change between 0 and 7 per week increase in GA at delivery; *β*_5_ is the difference in methylation change between 7 and 17 per week increase in GA. A random intercept-only model is reported, since random slope models could not be fitted to these data due to the small number of repeated measures. The models were further adjusted for the first 20 ISVA components (which account for variance due to changing cell type proportions and batch effects), maternal age, maternal alcohol consumption, maternal education, maternal smoking, parity and delivery method as potential confounders.

### Phenotypic differences in adolescence

To investigate whether observed birth characteristic-related differences in methylation were associated with longer term phenotypic differences, we identified a set of 10 developmental phenotypes measured during childhood and adolescence. These were height (cm) and weight (kg; measured at 17 years); leg length (cm; at 11 years); lean mass (g), fat mass (g), bone mass (g) and bone mineral density (g/cm^2^; 17 years); IQ (at 8 years); lung capacity [forced expiratory volume (l) in 1 second and forced vital capacity (l) at 15 years]. These outcomes were chosen to represent development in size (height, weight, leg length, lean/bone/fat mass and bone mineral density), mental function (IQ) and physical function (lung capacity) and based on published evidence that each has been associated with birth weight and/or GA (60–68). Using simple linear regression, these 10 phenotypes were tested for association with birth weight/GA as well as cord blood methylation from those CpG sites identified through EWAS. To account for multiple testing, evidence for association was set at the *P* < 0.05/(10 [phenotypes] × number of CpG sites).

## Supplementary Material

Supplementary Material is available at *HMG* online.

## Funding

This research was specifically funded by UK Economic & Social Research Council grant RES-060-23-0011, UK Medical Research Council grants G0601625, G0600705 and MR/L011824/1, and European Research Council grant 269874. Funding to pay the Open Access publication charges for this article was provided by The University of Bristol - RCUK Open Access fund.

## Supplementary Material

Supplementary Data

## References

[1] Irving R.J. Belton N.R. Elton R.A. Walker B.R. (2000) Adult cardiovascular risk factors in premature babies. The Lancet, 355, 2135–2136. 10.1016/S0140-6736(00)02384-9 10902631

[2] Behrman R.E. Butler A.S. (2007) Preterm Birth: Causes, Consequences, and Prevention. National Academies Press, Washington DC, USA. 20669423

[3] Aarnoudse-Moens C.S.H. Weisglas-Kuperus N. van Goudoever J.B. Oosterlaan J. (2009) Meta-analysis of neurobehavioral outcomes in very preterm and/or very low birth weight children. Pediatrics, 124, 717–728. 19651588 10.1542/peds.2008-2816

[4] Kwinta P. Pietrzyk J.J. (2010) Preterm birth and respiratory disease in later life. Expert Rev. Respir. Med., 4, 593–604. 20923339 10.1586/ers.10.59

[5] Schroeder J.W. Conneely K.N. Cubells J.C. Kilaru V. Newport D.J. Knight B.T. Stowe Z.N. Brennan P.A. Krushkal J. Tylavsky F.A. (2011) Neonatal DNA methylation patterns associate with gestational age. Epigenetics, 6, 1498–1504. 22139580 10.4161/epi.6.12.18296 PMC3256334

[6] Lee H. Jaffe A.E. Feinberg J.I. Tryggvadottir R. Brown S. Montano C. Aryee M.J. Irizarry R.A. Herbstman J. Witter F.R. (2012) DNA methylation shows genome-wide association of NFIX, RAPGEF2 and MSRB3 with gestational age at birth. Int. J. Epidemiol., 41, 188–199. 22422452 10.1093/ije/dyr237 PMC3304532

[7] Parets S.E. Conneely K.N. Kilaru V. Fortunato S.J. Syed T.A. Saade G. Smith A.K. Menon R. (2013) Fetal DNA methylation associates with early spontaneous preterm birth and gestational age. PLoS ONE, 8, e67489. 23826308 10.1371/journal.pone.0067489 PMC3694903

[8] Engel S.M. Joubert B.R. Wu M.C. Olshan A.F. Håberg S.E. Ueland P.M. Nystad W. Nilsen R.M. Vollset S.E. Peddada S.D. (2014) Neonatal genome-wide methylation patterns in relation to birth weight in the Norwegian Mother and Child Cohort. Am. J. Epidemiol., 179, 834–842. 24561991 10.1093/aje/kwt433 PMC3969535

[9] Gluckman P.D. Hanson M.A. Cooper C. Thornburg K.L. (2008) Effect of in utero and early-life conditions on adult health and disease. N. Engl. J. Med., 359, 61–73. 18596274 10.1056/NEJMra0708473 PMC3923653

[10] Gluckman P.D. Hanson M.A. Buklijas T. Low F.M. Beedle A.S. (2009) Epigenetic mechanisms that underpin metabolic and cardiovascular diseases. Nat. Rev. Endocr., 5, 401–408. 10.1038/nrendo.2009.102 19488075

[11] Heijmans B.T. Tobi E.W. Stein A.D. Putter H. Blauw G.J. Susser E.S. Slagboom P.E. Lumey L. (2008) Persistent epigenetic differences associated with prenatal exposure to famine in humans. Proc. Natl. Acad. Sci., 105, 17046–17049. 18955703 10.1073/pnas.0806560105 PMC2579375

[12] Kahn H.S. Graff M. Stein A.D. Lumey L. (2009) A fingerprint marker from early gestation associated with diabetes in middle age: the Dutch Hunger Winter Families Study. Int. J. Epidemiol., 38, 101–109. 18684786 10.1093/ije/dyn158 PMC2639363

[13] Tobi E.W. Lumey L. Talens R.P. Kremer D. Putter H. Stein A.D. Slagboom P.E. Heijmans B.T. (2009) DNA methylation differences after exposure to prenatal famine are common and timing-and sex-specific. Hum. Mol. Genet., 18, 4046–4053. 19656776 10.1093/hmg/ddp353 PMC2758137

[14] Whitson R.H. Tsark W. Huang T.H. Itakura K. (2003) Neonatal mortality and leanness in mice lacking the ARID transcription factor Mrf-2. Biochem. Biophys. Res. Commun., 312, 997–1004. 14651970 10.1016/j.bbrc.2003.11.026

[15] Pierce A.J. Johnson R.D. Thompson L.H. Jasin M. (1999) XRCC3 promotes homology-directed repair of DNA damage in mammalian cells. Genes Dev., 13, 2633–2638. 10541549 10.1101/gad.13.20.2633 PMC317094

[16] Griffin C.S. Simpson P.J. Wilson C.R. Thacker J. (2000) Mammalian recombination-repair genes XRCC2 and XRCC3 promote correct chromosome segregation. Nat. Cell Biol., 2, 757–761. 11025669 10.1038/35036399

[17] Tan Q. Frost M. Heijmans B.T. von Bornemann Hjelmborg J. Tobi E.W. Christensen K. Christiansen L. (2014) Epigenetic signature of birth weight discordance in adult twins. BMC Genomics, 15, 1062. 25476734 10.1186/1471-2164-15-1062 PMC4302120

[18] Novakovic B. Yuen R.K. Gordon L. Penaherrera M.S. Sharkey A. Moffett A. Craig J.M. Robinson W.P. Saffery R. (2011) Evidence for widespread changes in promoter methylation profile in human placenta in response to increasing gestational age and environmental/stochastic factors. BMC Genomics, 12, 529. 22032438 10.1186/1471-2164-12-529 PMC3216976

[19] Cruickshank M.N. Oshlack A. Theda C. Davis P.G. Martino D. Sheehan P. Dai Y. Saffery R. Doyle L.W. Craig J.M. (2013) Analysis of epigenetic changes in survivors of preterm birth reveals the effect of gestational age and evidence for a long term legacy. Genome Med., 5, 96. 24134860 10.1186/gm500 PMC3978871

[20] Jaenisch R. Bird A. (2003) Epigenetic regulation of gene expression: how the genome integrates intrinsic and environmental signals. Nat. Genet., 33, 245–254. 12610534 10.1038/ng1089

[21] Migliore L. Coppedè F. (2009) Genetics, environmental factors and the emerging role of epigenetics in neurodegenerative diseases. Mutat. Res.-Fund. Mol. Mech. M., 667, 82–97. 10.1016/j.mrfmmm.2008.10.011 19026668

[22] Feil R. Fraga M.F. (2012) Epigenetics and the environment: emerging patterns and implications. Nat. Rev. Gen., 13, 97–109. 10.1038/nrg3142 22215131

[23] Wong C. Caspi A. Williams B. Craig I.W. Houts R. Ambler A. Moffitt T.E. Mill J. (2010) A longitudinal study of epigenetic variation in twins. Epigenetics, 5, 516–526. 20505345 10.4161/epi.5.6.12226 PMC3322496

[24] Wang D. Liu X. Zhou Y. Xie H. Hong X. Tsai H.-J. Wang G. Liu R. Wang X. (2012) Individual variation and longitudinal pattern of genome-wide DNA methylation from birth to the first two years of life. Epigenetics, 7, 594–605. 22522910 10.4161/epi.20117 PMC3398988

[25] Madrigano J. Baccarelli A. Mittleman M.A. Sparrow D. Vokonas P.S. Tarantini L. Schwartz J. (2012) Aging and epigenetics. Epigenetics, 7, 63–70. 22207354 10.4161/epi.7.1.18749 PMC3329504

[26] Alisch R.S. Barwick B.G. Chopra P. Myrick L.K. Satten G.A. Conneely K.N. Warren S.T. (2012) Age-associated DNA methylation in pediatric populations. Genome Res., 22, 623–632. 22300631 10.1101/gr.125187.111 PMC3317145

[27] Bell J.T. Tsai P.-C. Yang T.-P. Pidsley R. Nisbet J. Glass D. Mangino M. Zhai G. Zhang F. Valdes A. (2012) Epigenome-wide scans identify differentially methylated regions for age and age-related phenotypes in a healthy ageing population. PLoS Genet., 8, e1002629. 22532803 10.1371/journal.pgen.1002629 PMC3330116

[28] Heyn H. Li N. Ferreira H.J. Moran S. Pisano D.G. Gomez A. Diez J. Sanchez-Mut J.V. Setien F. Carmona F.J. (2012) Distinct DNA methylomes of newborns and centenarians. Proc. Natl. Acad. Sci., 109, 10522–10527. 22689993 10.1073/pnas.1120658109 PMC3387108

[29] Johansson Å. Enroth S. Gyllensten U. (2013) Continuous aging of the human DNA methylome throughout the human lifespan. PLoS ONE, 8, e67378. 23826282 10.1371/journal.pone.0067378 PMC3695075

[30] Florath I. Butterbach K. Müller H. Bewerunge-Hudler M. Brenner H. (2013) Cross-sectional and longitudinal changes in DNA methylation with age: an epigenome-wide analysis revealing over 60 novel age-associated CpG sites. Hum. Mol. Genet., 23, 1186–1201. 24163245 10.1093/hmg/ddt531 PMC3919014

[31] Herbstman J.B. Wang S. Perera F.P. Lederman S.A. Vishnevetsky J. Rundle A.G. Hoepner L.A. Qu L. Tang D. (2013) Predictors and consequences of global DNA methylation in cord blood and at three years. PLoS ONE, 8, e72824. 24023780 10.1371/journal.pone.0072824 PMC3762861

[32] Hannum G. Guinney J. Zhao L. Zhang L. Hughes G. Sadda S. Klotzle B. Bibikova M. Fan J.-B. Gao Y. (2013) Genome-wide methylation profiles reveal quantitative views of human aging rates. Mol. Cell, 49, 359–367. 23177740 10.1016/j.molcel.2012.10.016 PMC3780611

[33] Martino D. Loke Y.J. Gordon L. Ollikainen M. Cruickshank M.N. Saffery R. Craig J.M. (2013) Longitudinal, genome-scale analysis of DNA methylation in twins from birth to 18 months of age reveals rapid epigenetic change in early life and pair-specific effects of discordance. Genome Biol., 14, R42. 23697701 10.1186/gb-2013-14-5-r42 PMC4054827

[34] Martino D. Maksimovic J. Joo J.-H. Prescott S. Saffery R. (2012) Genome-scale profiling reveals a subset of genes regulated by DNA methylation that program somatic T-cell phenotypes in humans. Genes Immun., 13, 388–398. 22495533 10.1038/gene.2012.7

[35] Dedeurwaerder S. Defrance M. Calonne E. Denis H. Sotiriou C. Fuks F. (2011) Evaluation of the Infinium Methylation 450 K technology. Epigenomics, 3, 771–784. 22126295 10.2217/epi.11.105

[36] Houseman E.A. Molitor J. Marsit C.J. (2014) Reference-free cell mixture adjustments in analysis of DNA methylation data. Bioinformatics, 30, 1431–1439. 24451622 10.1093/bioinformatics/btu029 PMC4016702

[37] Magnus P. Irgens L.M. Haug K. Nystad W. Skjærven R. Stoltenberg C. (2006) Cohort profile: the Norwegian mother and child cohort study (MoBa). Int. J. Epidemiol., 35, 1146–1150. 16926217 10.1093/ije/dyl170

[38] Lee K. Richmond R. Hu P. French L. Shin J. Bourdon C. Reischl E. Waldenberger M. Zeilinger S. Gaunt T. (2014) Prenatal exposure to maternal cigarette smoking and DNA methylation: epigenome-wide association in a discovery sample of adolescents and replication in an independent cohort at birth through 17 years of age. Environ. Health Perspect, 123, 193–199. 25325234 10.1289/ehp.1408614 PMC4314251

[39] Richmond R.C. Simpkin A.J. Woodward G. Gaunt T.R. Lyttleton O. McArdle W.L. Ring S.M. Smith A.D. Timpson N.J. Tilling K. (2014) Prenatal exposure to maternal smoking and offspring DNA methylation across the lifecourse: findings from the Avon Longitudinal Study of Parents and Children (ALSPAC). Hum. Mol. Genet., 24, 2201–2217. 25552657 10.1093/hmg/ddu739 PMC4380069

[40] Sharp G. Lawlor D. Richmond R. Fraser A. Simpkin A. Suderman M. Shihab H. Lyttleton O. McArdle W. Ring S. (2015) Maternal pre-pregnancy BMI and gestational weight gain, offspring DNA methylation and later offspring adiposity: findings from the Avon Longitudinal Study of Parents and Children. Int. J. Epidemiol., doi:10.1093/ije/dyv042. 10.1093/ije/dyv042 PMC4588865 25855720

[41] Shields J.D. Kourtis I.C. Tomei A.A. Roberts J.M. Swartz M.A. (2010) Induction of lymphoid-like stroma and immune escape by tumors that express the chemokine CCL21. Science, 328, 749–752. 20339029 10.1126/science.1185837

[42] Harvey N.C. Lillycrop K.A. Garratt E. Sheppard A. McLean C. Burdge G. Slater-Jefferies J. Rodford J. Crozier S. Inskip H. (2012) Evaluation of methylation status of the eNOS promoter at birth in relation to childhood bone mineral content. Calcif. Tissue Int., 90, 120–127. 22159788 10.1007/s00223-011-9554-5 PMC3629299

[43] Åkerlund M. (2002) Involvement of oxytocin and vasopressin in the pathophysiology of preterm labor and primary dysmenorrhea. Prog. Brain Res., 139, 359–365. 12436949 10.1016/s0079-6123(02)39030-7

[44] Salomonis N. Cotte N. Zambon A.C. Pollard K.S. Vranizan K. Doniger S.W. Dolganov G. Conklin B.R. (2005) Identifying genetic networks underlying myometrial transition to labor. Genome Biol., 6, R12. 15693941 10.1186/gb-2005-6-2-r12 PMC551532

[45] Stadtman E.R. Van Remmen H. Richardson A. Wehr N.B. Levine R.L. (2005) Methionine oxidation and aging. Biochim. Biophys. Acta, 1703, 135–140. 15680221 10.1016/j.bbapap.2004.08.010

[46] Shringarpure R. Davies K.J. (2002) Protein turnover by the proteasome in aging and disease. Free Radic. Biol. Med., 32, 1084–1089. 12031893 10.1016/s0891-5849(02)00824-9

[47] Van Engelen E. Breeveld-Dwarkasing V. Taverne M. Everts M. Van der Weijden G. Rutten V. (2008) MMP-2 expression precedes the final ripening process of the bovine cervix. Mol. Reprod. Dev., 75, 1669–1677. 18361420 10.1002/mrd.20908

[48] Lufkin T. Lohnes D. Mark M. Dierich A. Gorry P. Gaub M.-P. LeMeur M. Chambon P. (1993) High postnatal lethality and testis degeneration in retinoic acid receptor alpha mutant mice. Proc. Natl. Acad. Sci., 90, 7225–7229. 8394014 10.1073/pnas.90.15.7225 PMC47109

[49] Messina G. Biressi S. Monteverde S. Magli A. Cassano M. Perani L. Roncaglia E. Tagliafico E. Starnes L. Campbell C.E. (2010) Nfix regulates fetal-specific transcription in developing skeletal muscle. Cell, 140, 554–566. 20178747 10.1016/j.cell.2010.01.027

[50] Randall T.D. Carragher D.M. Rangel-Moreno J. (2008) Development of secondary lymphoid organs. Annu. Rev. Immunol., 26, 627. 18370924 10.1146/annurev.immunol.26.021607.090257 PMC2590644

[51] Trainor P.A. Krumlauf R. (2000) Patterning the cranial neural crest: Hinbrain segmentation and hox gene plasticity. Nat. Rev. Neurosci. 1, 116–124. 11252774 10.1038/35039056

[52] Durbán M. Harezlak J. Wand M. Carroll R. (2005) Simple fitting of subject-specific curves for longitudinal data. Stat. Med., 24, 1153–1167. 15568201 10.1002/sim.1991

[53] Fraser A. Macdonald-Wallis C. Tilling K. Boyd A. Golding J. Smith G.D. Henderson J. Macleod J. Molloy L. Ness A. (2013) Cohort profile: the Avon Longitudinal Study of Parents and Children: ALSPAC mothers cohort. Int. J. Epidemiol., 42, 97–110. 22507742 10.1093/ije/dys066 PMC3600619

[54] Touleimat N. Tost J. (2012) Complete pipeline for Infinium^®^ Human Methylation 450 K BeadChip data processing using subset quantile normalization for accurate DNA methylation estimation. Epigenomics, 4, 325–341. 22690668 10.2217/epi.12.21

[55] Barfield R.T. Kilaru V. Smith A.K. Conneely K.N. (2012) CpGassoc: an R function for analysis of DNA methylation microarray data. Bioinformatics, 28, 1280–1281. 22451269 10.1093/bioinformatics/bts124 PMC3577110

[56] Du P. Zhang X. Huang C.-C. Jafari N. Kibbe W.A. Hou L. Lin S.M. (2010) Comparison of Beta-value and M-value methods for quantifying methylation levels by microarray analysis. BMC Bioinformatics, 11, 587. 21118553 10.1186/1471-2105-11-587 PMC3012676

[57] Teschendorff A.E. Zhuang J. Widschwendter M. (2011) Independent surrogate variable analysis to deconvolve confounding factors in large-scale microarray profiling studies. Bioinformatics, 27, 1496–1505. 21471010 10.1093/bioinformatics/btr171

[58] Laird N.M. Ware J.H. (1982) Random-effects models for longitudinal data. Biometrics, 38, 963–974. 7168798

[59] Goldstein H. (1986) Multilevel mixed linear-model analysis using iterative generalized least-squares. Biometrika, 73, 43–56.

[60] Lawlor D.A. Davey Smith G. Ebrahim S. (2003) Association between leg length and offspring birthweight: partial explanation for the trans-generational association between birthweight and cardiovascular disease: findings from the British Women's Heart and Health Study. Paediatr. Perinat. Epidemiol., 17, 148–155. 12675781 10.1046/j.1365-3016.2003.00479.x

[61] Lawlor D.A. Ebrahim S. Smith G.D. (2005) Association of birth weight with adult lung function: findings from the British Women's Heart and Health Study and a meta-analysis. Thorax, 60, 851–858. 16055617 10.1136/thx.2005.042408 PMC1747204

[62] Sørensen H.T. Sabroe S. Rothman K.J. Gillman M. Steffensen F.H. Fischer P. Serensen T.I. (1999) Birth weight and length as predictors for adult height. Am. J. Epidemiol., 149, 726–729. 10206622 10.1093/oxfordjournals.aje.a009881

[63] Eide M.G. Øyen N. Skjærven R. Nilsen S.T. Bjerkedal T. Tell G.S. (2005) Size at birth and gestational age as predictors of adult height and weight. Epidemiology, 16, 175–181. 15703531 10.1097/01.ede.0000152524.89074.bf

[64] Yarbrough D. Barrett-Connor E. Morton D. (2000) Birth weight as a predictor of adult bone mass in postmenopausal women: the Rancho Bernardo Study. Osteoporos. Int., 11, 626–630. 11069198 10.1007/s001980070085

[65] Singhal A. Wells J. Cole T.J. Fewtrell M. Lucas A. (2003) Programming of lean body mass: a link between birth weight, obesity, and cardiovascular disease? Am. J. Clin. Nutr., 77, 726–730. 12600868 10.1093/ajcn/77.3.726

[66] Hamed H. Purdie D. Ramsden C. Carmichael B. Steel S. Howey S. (1993) Influence of birth weight on adult bone mineral density. Osteoporos. Int., 3, 1–2. 8422509 10.1007/BF01623168

[67] Barker M. Robinson S. Osmond C. Barker D. (1997) Birth weight and body fat distribution in adolescent girls. Arch. Dis. Child., 77, 381–383. 9487954 10.1136/adc.77.5.381

[68] Matte T.D. Bresnahan M. Begg M.D. Susser E. (2001) Influence of variation in birth weight within normal range and within sibships on IQ at age 7 years: cohort study. BMJ, 323, 310–314. 11498487 10.1136/bmj.323.7308.310 PMC37317

