# Supplementary material for: Longitudinal analysis of DNA methylation associated with birth weight and gestational age
Source: Hum Mol Genet. 2015 Apr 13;24(13):3752–63. doi: 10.1093/hmg/ddv119 (PMC4459393; doi:10.1093/hmg/ddv119)
Supplement: Supplementary Data [file supp_24_13_3752__index.html]

Longitudinal analysis of DNA methylation associated with birth weight and gestational age — Longitudinal analysis of DNA methylation associated with birth weight and gestational age — Longitudinal analysis of DNA methylation associated with birth weight and gestational age — Supplementary Data 

# Longitudinal analysis of DNA methylation associated with birth weight and gestational age

## Supplementary Data

Supplementary Data

**Files in this Data Supplement:**

- Supplementary Data - Docx file
