# Supplementary material for: Longitudinal analysis of DNA methylation associated with birth weight and gestational age
Source: Hum Mol Genet. 2015 Apr 13;24(13):3752–63. doi: 10.1093/hmg/ddv119 (PMC4459393; doi:10.1093/hmg/ddv119)
Supplement: Supplementary Data [file supp_ddv119_ddv119supp.docx]

Table S1: Probes at which cord blood methylation is associated with gestational age in ARIES cohort only

|  | EWAS Results | | | | | | Longitudinal analysis results | | | |
| --- | --- | --- | --- | --- | --- | --- | --- | --- | --- | --- |
| Probe | **Associated gene** | **Chr** | **Coordinates** | **t-statistic** | **p-value** | **FDR p-value** | **Estimated cord blood methylation (%)** | **Average yearly change**  **between 0-7 (%)** | **Average yearly change**  **between 7-17 (%)** | **Average difference in childhood yearly change per week increase in GA at delivery (%)** |
| Probes with negative association between gestational age and methylation in the ARIES cohort only | | | | | | | | | | |
| cg08943494* | *PRR5L* | 11 | 36379191 | -11.97 | 3.00E-30 | 1.50E-24 | 60.0 | -4.7 | n/a | 0.37 |
| cg11932158* | *PLCH1* | 3 | 156904823 | -10.54 | 2.90E-24 | 7.00E-19 | 70.4 | -5.4 | -0.15 | 0.23 |
| cg16725984* | *C16orf55* | 16 | 88262685 | -10.39 | 1.20E-23 | 1.90E-18 | 67.3 | -4.4 | 0.12 | 0.34 |
| cg20334115* | *PYCR2* | 1 | 224174522 | -9.89 | 1.00E-21 | 1.30E-16 | 65.8 | -3.5 | n/a | 0.21 |
| cg18623216* | *PLCH1* | 3 | 156904664 | -9.69 | 5.80E-21 | 5.70E-16 | 63.7 | -4.8 | -0.21 | 0.25 |
| cg07835443* | *C16orf55* | 16 | 88262487 | -9.28 | 2.00E-19 | 1.70E-14 | 43.3 | -2.5 | 0.12 | 0.33 |
| cg00220721* | *PRR5L* | 11 | 36379019 | -9.12 | 7.60E-19 | 5.30E-14 | 58.3 | -4.3 | n/a | 0.26 |
| cg04685228 | *N/A* | 5 | 172395232 | -8.94 | 3.20E-18 | 1.90E-13 | 55.0 | -5.7 | n/a | 0.22 |
| cg16103712* | *MATN2* | 8 | 99093045 | -8.75 | 1.60E-17 | 8.50E-13 | 56.5 | -4.2 | -0.13 | 0.26 |
| cg27518892* | *CCDC102A* | 16 | 56124437 | -8.69 | 2.40E-17 | 1.10E-12 | 39.0 | -1.9 | 0.11 | 0.15 |
| cg22117805 | *PRR5L* | 11 | 36379031 | -8.62 | 4.30E-17 | 1.70E-12 | 59.9 | -5.0 | -0.24 | 0.29 |
| cg21926626* | *TSNARE1* | 8 | 143380133 | -8.54 | 7.90E-17 | 2.90E-12 | 74.2 | -3.2 | n/a | 0.20 |
| cg04347477* | *NCOR2* | 12 | 123567960 | -8.47 | 1.40E-16 | 4.80E-12 | 71.0 | -2.6 | n/a | 0.20 |
| cg08817867* | *N/A* | 17 | 19597146 | -8.30 | 5.00E-16 | 1.60E-11 | 44.2 | -2.0 | n/a | 0.24 |
| cg27448161 | *SPRYD3* | 12 | 51759059 | -8.20 | 1.10E-15 | 3.30E-11 | 39.1 | -2.4 | n/a | 0.08 |
| cg01154283 | *CRIM1* | 2 | 36457047 | -8.12 | 2.00E-15 | 5.60E-11 | 67.9 | -2.0 | -0.20 | 0.14 |
| cg02001279* | *ARID3A* | 19 | 891967 | -8.11 | 2.20E-15 | 5.90E-11 | 59.1 | -4.2 | -0.37 | 0.23 |
| cg02430430* | *CCDC33* | 15 | 72319503 | -7.99 | 5.30E-15 | 1.30E-10 | 53.2 | -1.9 | -0.20 | 0.20 |
| cg06870470* | *DOCK6* | 19 | 11176767 | -7.95 | 7.00E-15 | 1.70E-10 | 36.1 | -3.5 | n/a | 0.29 |
| cg13924996 | *ADRBK1* | 11 | 66810405 | -7.87 | 1.30E-14 | 3.00E-10 | 63.9 | -3.2 | -0.62 | 0.18 |
| cg07136133 | *PRR5L* | 11 | 36378953 | -7.80 | 2.20E-14 | 4.90E-10 | 54.9 | -3.6 | -0.26 | 0.25 |
| cg00481600 | *CILP* | 15 | 63290882 | -7.73 | 3.70E-14 | 7.80E-10 | 55.9 | -1.9 | -0.15 | 0.10 |
| cg13675859 | *GPR110* | 6 | 47110932 | -7.72 | 3.90E-14 | 7.80E-10 | 74.2 | -1.3 | -0.19 | 0.17 |
| cg11934771* | *HCG22* | 6 | 31129775 | -7.67 | 5.80E-14 | 1.10E-09 | 33.0 | -1.9 | n/a | 0.14 |
| cg19744173* | *FBLN7* | 2 | 112629649 | -7.63 | 7.60E-14 | 1.40E-09 | 40.0 | -2.0 | -0.11 | 0.14 |
| cg12713583 | *ARID3A* | 19 | 891724 | -7.55 | 1.30E-13 | 2.30E-09 | 64.8 | -6.8 | -0.14 | 0.28 |
| cg11360522* | *ATP11A* | 13 | 112427829 | -7.43 | 3.00E-13 | 5.20E-09 | 44.2 | -2.5 | 0.18 | 0.13 |
| cg16834726 | *DCTN2* | 12 | 56223675 | -7.41 | 3.60E-13 | 6.10E-09 | 66.8 | -2.2 | -0.24 | 0.14 |
| cg18608055 | *SBNO2* | 19 | 1081866 | -7.24 | 1.10E-12 | 1.80E-08 | 62.0 | -1.2 | 0.26 | 0.11 |
| cg05283597* | *ITIH1* | 3 | 52787450 | -7.22 | 1.30E-12 | 2.10E-08 | 65.6 | -2.9 | -0.26 | 0.17 |
| cg08726900 | *ANKRD11* | 16 | 88077975 | -7.16 | 2.00E-12 | 3.00E-08 | 45.1 | -2.3 | n/a | 0.21 |
| cg00166343 | *CRLF3* | 17 | 26174226 | -7.11 | 2.80E-12 | 4.20E-08 | 21.2 | -1.1 | n/a | 0.15 |
| cg10239319 | *JAKMIP1* | 4 | 6124557 | -7.06 | 3.80E-12 | 5.40E-08 | 65.1 | -2.5 | n/a | 0.15 |
| cg24024661 | *HMHA1* | 19 | 1025425 | -7.05 | 4.20E-12 | 5.90E-08 | 69.7 | -4.6 | -0.51 | 0.13 |
| cg08645860 | *N/A* | 16 | 84526802 | -7.01 | 5.40E-12 | 7.30E-08 | 54.2 | -0.7 | n/a | 0.16 |
| cg21081878* | *HLCS* | 21 | 37256600 | -6.98 | 6.70E-12 | 8.80E-08 | 55.9 | 0.8 | 0.44 | 0.16 |
| cg04632887* | *AVP* | 20 | 3013559 | -6.91 | 1.10E-11 | 1.40E-07 | 72.2 | -3.9 | n/a | 0.17 |
| cg11854981 | *TRERF1* | 6 | 42327825 | -6.90 | 1.10E-11 | 1.40E-07 | 69.2 | -2.8 | -0.39 | 0.19 |
| cg24741609 | *GLIS1* | 1 | 53894648 | -6.90 | 1.10E-11 | 1.40E-07 | 26.4 | 1.0 | 0.25 | 0.09 |
| cg04494800 | *ZC3H12D* | 6 | 149817546 | -6.88 | 1.30E-11 | 1.50E-07 | 66.9 | -3.2 | -0.40 | 0.13 |
| cg27295118 | *N/A* | 14 | 21972066 | -6.84 | 1.60E-11 | 1.90E-07 | 69.3 | -5.8 | n/a | 0.18 |
| cg21132686* | *N/A* | 20 | 22743774 | -6.81 | 2.00E-11 | 2.20E-07 | 37.3 | -1.7 | 0.16 | 0.15 |
| cg03290131 | *DUSP5* | 10 | 112253821 | -6.81 | 2.10E-11 | 2.20E-07 | 35.9 | -1.1 | -0.42 | 0.16 |
| cg05969150* | *ZFHX3* | 16 | 71439996 | -6.81 | 2.10E-11 | 2.20E-07 | 65.6 | -3.4 | -0.23 | 0.12 |
| cg23062810* | *CLIP2* | 7 | 73358744 | -6.78 | 2.60E-11 | 2.70E-07 | 45.8 | -4.2 | -0.22 | 0.15 |
| cg03940776 | *SYNJ2* | 6 | 158410001 | -6.77 | 2.70E-11 | 2.80E-07 | 57.5 | -1.2 | -0.17 | 0.11 |
| cg07810039 | *TGFB2* | 1 | 216591181 | -6.70 | 4.20E-11 | 4.20E-07 | 68.4 | -7.0 | -0.16 | 0.15 |
| cg17477052* | *COL13A1* | 10 | 71266737 | -6.67 | 5.00E-11 | 4.90E-07 | 52.6 | -3.0 | -0.18 | 0.19 |
| cg26170244 | *N/A* | 15 | 62963406 | -6.63 | 6.40E-11 | 6.10E-07 | 47.7 | -1.3 | -0.53 | 0.12 |
| cg06442489 | *ZSCAN18* | 19 | 63322424 | -6.62 | 7.20E-11 | 6.70E-07 | 77.4 | -0.8 | -0.25 | 0.16 |
| cg00153101* | *PLCH2* | 1 | 2421613 | -6.59 | 8.40E-11 | 7.60E-07 | 36.0 | n/a | 0.09 | 0.09 |
| cg26027170 | *N/A* | 17 | 34648231 | -6.59 | 8.60E-11 | 7.60E-07 | 37.5 | -2.7 | -0.12 | 0.14 |
| cg04416898 | *CXXC5* | 5 | 139020239 | -6.55 | 1.10E-10 | 9.10E-07 | 37.9 | n/a | -0.36 | 0.18 |
| cg03048432 | *NIN* | 14 | 50360501 | -6.55 | 1.10E-10 | 9.10E-07 | 70.6 | -2.9 | n/a | 0.12 |
| cg01229865 | *ATP11A* | 13 | 112576752 | -6.54 | 1.10E-10 | 9.60E-07 | 69.3 | -4.3 | -0.28 | 0.13 |
| cg12697139 | *N/A* | 1 | 207638512 | -6.53 | 1.20E-10 | 1.00E-06 | 53.4 | -5.0 | -0.20 | 0.26 |
| cg11873462 | *ZC3H3* | 8 | 144683083 | -6.51 | 1.40E-10 | 1.20E-06 | 55.5 | -2.6 | n/a | 0.19 |
| cg07173823 | *C1orf228* | 1 | 44963106 | -6.48 | 1.70E-10 | 1.40E-06 | 57.1 | -1.1 | -0.24 | 0.13 |
| cg08620426* | *N/A* | 16 | 29514531 | -6.48 | 1.80E-10 | 1.40E-06 | 48.0 | -3.1 | 0.13 | 0.11 |
| cg12758082* | *ATP11A* | 13 | 112455770 | -6.47 | 1.80E-10 | 1.40E-06 | 44.2 | -1.4 | n/a | n/a |
| cg02753354* | *HMHA1* | 19 | 1025727 | -6.47 | 1.80E-10 | 1.40E-06 | 21.3 | -1.6 | n/a | 0.13 |
| cg05820066 | *LDHD* | 16 | 73703344 | -6.46 | 2.00E-10 | 1.50E-06 | 59.6 | -1.3 | -0.23 | 0.12 |
| cg16536330 | *LEPROTL1* | 8 | 30076886 | -6.45 | 2.00E-10 | 1.50E-06 | 18.1 | -0.5 | -0.21 | 0.11 |
| cg07749613* | *N/A* | 2 | 96437266 | -6.43 | 2.40E-10 | 1.70E-06 | 12.8 | -0.9 | n/a | 0.11 |
| cg13283635 | *COL9A3* | 20 | 60921098 | -6.42 | 2.40E-10 | 1.80E-06 | 62.1 | -2.1 | -0.18 | 0.15 |
| cg08282819* | *IL21R* | 16 | 27345401 | -6.39 | 2.90E-10 | 2.00E-06 | 28.7 | -3.0 | n/a | 0.24 |
| cg21223094 | *ETV6* | 12 | 11866782 | -6.39 | 3.00E-10 | 2.00E-06 | 44.6 | -2.5 | n/a | 0.16 |
| cg18598117* | *ARID3A* | 19 | 892126 | -6.39 | 3.00E-10 | 2.00E-06 | 82.0 | -6.1 | -0.66 | n/a |
| cg10020892 | *BCL9* | 1 | 145483525 | -6.37 | 3.40E-10 | 2.30E-06 | 18.3 | -1.4 | n/a | 0.16 |
| cg08915038* | *C1orf89* | 1 | 16435510 | -6.33 | 4.20E-10 | 2.80E-06 | 16.4 | -1.1 | n/a | 0.11 |
| cg15030415* | *RAP1GAP2* | 17 | 2854818 | -6.32 | 4.50E-10 | 2.90E-06 | 67.8 | -1.9 | -0.23 | 0.15 |
| cg14656043 | *CREM* | 10 | 35460002 | -6.32 | 4.50E-10 | 2.90E-06 | 42.2 | -1.6 | n/a | 0.15 |
| cg01394781 | *ABCC1* | 16 | 16121714 | -6.27 | 6.10E-10 | 3.80E-06 | 64.8 | -1.8 | -0.20 | 0.15 |
| cg13027206 | *CCDC88C* | 14 | 90936078 | -6.27 | 6.20E-10 | 3.80E-06 | 34.1 | -2.4 | n/a | 0.08 |
| cg00360761 | *N/A* | 2 | 43251583 | -6.27 | 6.30E-10 | 3.80E-06 | 38.2 | -0.6 | -0.41 | 0.11 |
| cg21121843 | *HTT* | 4 | 3173780 | -6.25 | 7.00E-10 | 4.20E-06 | 59.1 | -2.2 | -0.24 | 0.17 |
| cg07813031 | *N/A* | 1 | 61071009 | -6.25 | 7.00E-10 | 4.20E-06 | 63.4 | -6.3 | -0.29 | 0.16 |
| cg07573872 | *SBNO2* | 19 | 1077342 | -6.25 | 7.20E-10 | 4.20E-06 | 76.8 | -0.2 | -0.12 | 0.16 |
| cg23169111 | *AVP* | 20 | 3013582 | -6.24 | 7.30E-10 | 4.20E-06 | 58.8 | -2.2 | n/a | 0.07 |
| cg07969918 | *PIK3IP1* | 22 | 30012909 | -6.22 | 8.50E-10 | 4.90E-06 | 45.5 | -0.8 | -0.22 | n/a |
| cg27471246* | *ERG* | 21 | 38698304 | -6.20 | 9.30E-10 | 5.30E-06 | 46.3 | -1.3 | -0.16 | 0.11 |
| cg04902474 | *KIAA1522* | 1 | 33006060 | -6.19 | 1.00E-09 | 5.60E-06 | 60.1 | -2.3 | -0.16 | 0.10 |
| cg18520238 | *PRR5L* | 11 | 36379133 | -6.18 | 1.10E-09 | 6.00E-06 | 36.2 | -3.4 | n/a | 0.21 |
| cg24914185 | *N/A* | 2 | 200372034 | -6.17 | 1.10E-09 | 6.10E-06 | 75.3 | -3.1 | -0.20 | n/a |
| cg02983090* | *IL21R* | 16 | 27345393 | -6.16 | 1.20E-09 | 6.40E-06 | 35.9 | -3.5 | n/a | 0.24 |
| cg00442282* | *RARA* | 17 | 35724590 | -6.15 | 1.30E-09 | 6.50E-06 | 26.1 | -1.2 | -0.08 | 0.12 |
| cg12170787* | *SBNO2* | 19 | 1081965 | -6.15 | 1.30E-09 | 6.50E-06 | 56.5 | -1.0 | 0.28 | 0.09 |
| cg19136673* | *PSMB8* | 6 | 32921315 | -6.15 | 1.30E-09 | 6.50E-06 | 34.7 | -2.7 | n/a | 0.08 |
| cg10535320* | *PSMC5* | 17 | 59262601 | -6.14 | 1.40E-09 | 6.80E-06 | 53.4 | -0.9 | -0.17 | 0.10 |
| cg21374307 | *SCHIP1* | 3 | 161030233 | -6.12 | 1.50E-09 | 7.70E-06 | 64.1 | -0.7 | -0.49 | 0.24 |
| cg18681014 | *N/A* | 14 | 104731921 | -6.09 | 1.90E-09 | 9.10E-06 | 71.2 | -3.5 | -0.23 | 0.09 |
| cg21691065* | *N/A* | 1 | 2794695 | -6.07 | 2.00E-09 | 9.80E-06 | 57.9 | -4.2 | n/a | n/a |
| cg18157896 | *C20orf141;LOC100288797* | 20 | 2743593 | -6.06 | 2.20E-09 | 1.10E-05 | 71.7 | -3.4 | -0.23 | n/a |
| cg22828383* | *NCOR2* | 12 | 123568144 | -6.05 | 2.30E-09 | 1.10E-05 | 56.3 | -2.0 | n/a | 0.14 |
| cg11436362 | *ADRBK1* | 11 | 66810505 | -6.03 | 2.60E-09 | 1.20E-05 | 52.2 | -2.8 | -0.61 | 0.19 |
| cg19592472* | *OXT;OXT* | 20 | 3000274 | -6.03 | 2.60E-09 | 1.20E-05 | 85.6 | -1.8 | -0.40 | 0.08 |
| cg16936953 | *TMEM49* | 17 | 55270447 | -6.03 | 2.60E-09 | 1.20E-05 | 53.1 | n/a | n/a | 0.17 |
| cg24159436 | *PLCL2* | 3 | 16949685 | -6.01 | 3.00E-09 | 1.30E-05 | 71.8 | -3.0 | n/a | 0.10 |
| cg25124966 | *TMEM45B* | 11 | 129229197 | -5.99 | 3.30E-09 | 1.50E-05 | 25.5 | -0.9 | -0.27 | 0.11 |
| cg16356456* | *CLIP2* | 7 | 73358806 | -5.98 | 3.60E-09 | 1.60E-05 | 54.0 | -4.1 | -0.20 | 0.13 |
| cg25938530 | *ITIH1* | 3 | 52787407 | -5.97 | 3.70E-09 | 1.60E-05 | 67.6 | -3.6 | -0.29 | 0.18 |
| cg03047995* | *FITM1* | 14 | 23671428 | -5.96 | 3.80E-09 | 1.60E-05 | 39.0 | -2.4 | 0.27 | 0.15 |
| cg01787084 | *FBXO31* | 16 | 85928598 | -5.94 | 4.30E-09 | 1.80E-05 | 48.4 | -2.0 | 0.35 | 0.27 |
| cg24296397* | *BSN* | 3 | 49667541 | -5.93 | 4.80E-09 | 2.00E-05 | 70.1 | -3.6 | 0.36 | 0.13 |
| cg16767506* | *N/A* | 7 | 142204372 | -5.90 | 5.70E-09 | 2.40E-05 | 44.3 | 0.9 | 0.27 | 0.15 |
| cg11551448 | *CASP10* | 2 | 201768953 | -5.89 | 6.00E-09 | 2.40E-05 | 70.5 | 0.6 | -0.19 | 0.10 |
| cg02571436 | *N/A* | 10 | 77138343 | -5.87 | 6.60E-09 | 2.60E-05 | 79.8 | -1.9 | 0.11 | 0.08 |
| cg27504369 | *APOBEC3A* | 22 | 37683496 | -5.85 | 7.40E-09 | 2.90E-05 | 50.8 | -3.4 | -0.30 | 0.16 |
| cg04411893 | *N/A* | 3 | 186783403 | -5.84 | 7.80E-09 | 3.00E-05 | 26.8 | -1.3 | -0.12 | 0.10 |
| cg19723805 | *N/A* | 5 | 33183296 | -5.83 | 8.40E-09 | 3.30E-05 | 70.5 | -3.1 | -0.31 | 0.14 |
| cg03720100 | *N/A* | 6 | 30828242 | -5.82 | 8.80E-09 | 3.40E-05 | 42.4 | -0.4 | -0.37 | 0.19 |
| cg02889774 | *XPO7* | 8 | 21880501 | -5.81 | 9.50E-09 | 3.60E-05 | 66.4 | -1.7 | n/a | n/a |
| cg14434062 | *N/A* | 15 | 68554473 | -5.80 | 1.00E-08 | 3.80E-05 | 38.7 | -2.3 | -0.23 | 0.17 |
| cg03699843 | *SNX20* | 16 | 49258565 | -5.80 | 1.00E-08 | 3.80E-05 | 30.2 | -0.8 | -0.29 | 0.14 |
| cg02747950 | *RAB8B* | 15 | 61313936 | -5.79 | 1.00E-08 | 3.80E-05 | 69.0 | -3.9 | -0.24 | n/a |
| cg05298628 | *VPS37B* | 12 | 121945522 | -5.79 | 1.00E-08 | 3.80E-05 | 31.9 | -0.9 | n/a | 0.10 |
| cg11832281 | *CUGBP2* | 10 | 11251028 | -5.79 | 1.10E-08 | 3.90E-05 | 17.6 | -0.5 | -0.29 | 0.10 |
| cg19913563 | *N/A* | 6 | 30828240 | -5.78 | 1.10E-08 | 4.00E-05 | 65.5 | -3.7 | -0.37 | 0.18 |
| cg10714639* | *HMHA1* | 19 | 1026104 | -5.78 | 1.10E-08 | 4.00E-05 | 42.1 | -3.6 | -0.16 | 0.12 |
| cg21155834 | *N/A* | 2 | 148998679 | -5.77 | 1.20E-08 | 4.00E-05 | 16.5 | -0.9 | n/a | 0.14 |
| cg16151538 | *TRPC4AP* | 20 | 33140049 | -5.76 | 1.20E-08 | 4.30E-05 | 72.5 | -1.0 | -0.22 | 0.13 |
| cg08745334 | *N/A* | 4 | 1723867 | -5.76 | 1.30E-08 | 4.30E-05 | 70.3 | -0.8 | n/a | 0.07 |
| cg17178761 | *MSI2* | 17 | 53037850 | -5.75 | 1.30E-08 | 4.50E-05 | 27.8 | -0.9 | 0.21 | n/a |
| cg05660874* | *SPPL2B* | 19 | 2283485 | -5.75 | 1.40E-08 | 4.60E-05 | 48.7 | -1.8 | 0.13 | n/a |
| cg08412913 | *N/A* | 16 | 83987023 | -5.74 | 1.40E-08 | 4.60E-05 | 41.1 | -1.9 | -0.16 | 0.11 |
| cg10493186 | *PRDM16* | 1 | 3124616 | -5.74 | 1.40E-08 | 4.60E-05 | 76.8 | -5.3 | -0.34 | 0.10 |
| cg24736933 | *C15orf52* | 15 | 38420586 | -5.73 | 1.50E-08 | 4.90E-05 | 56.8 | -2.5 | -0.66 | 0.16 |
| cg21559223 | *ATP2A3* | 17 | 3785463 | -5.71 | 1.60E-08 | 5.20E-05 | 27.5 | n/a | n/a | n/a |
| cg16847428 | *N/A* | 4 | 25704536 | -5.70 | 1.70E-08 | 5.50E-05 | 64.0 | 0.5 | -0.63 | 0.14 |
| cg20068209 | *TMEM30A* | 6 | 76045288 | -5.70 | 1.80E-08 | 5.60E-05 | 55.8 | -5.2 | -0.34 | 0.17 |
| cg24387542* | *LGALS9B* | 17 | 20311189 | -5.69 | 1.80E-08 | 5.60E-05 | 15.7 | -0.7 | n/a | 0.08 |
| cg23378033 | *MSRB3* | 12 | 63958298 | -5.69 | 1.90E-08 | 5.80E-05 | 58.7 | -1.8 | -0.35 | 0.26 |
| cg19439123* | *RAI1* | 17 | 17628346 | -5.66 | 2.10E-08 | 6.60E-05 | 59.8 | -2.5 | n/a | 0.17 |
| cg20454518* | *FBRSL1* | 12 | 131645536 | -5.65 | 2.30E-08 | 6.80E-05 | 56.1 | -1.5 | 0.12 | 0.07 |
| cg09163035 | *LASP1* | 17 | 34308252 | -5.65 | 2.30E-08 | 6.80E-05 | 28.1 | -1.6 | n/a | n/a |
| cg08161325 | *N/A* | 16 | 86680242 | -5.65 | 2.30E-08 | 6.80E-05 | 87.0 | -1.6 | -0.32 | 0.08 |
| cg08813888 | *N/A* | 2 | 652396 | -5.64 | 2.50E-08 | 7.30E-05 | 48.9 | -1.9 | n/a | 0.13 |
| cg22761205* | *PTDSS2* | 11 | 447256 | -5.63 | 2.50E-08 | 7.50E-05 | 41.8 | -1.4 | n/a | 0.10 |
| cg19265300* | *N/A* | 15 | 67489756 | -5.62 | 2.80E-08 | 8.10E-05 | 54.4 | -1.8 | n/a | 0.14 |
| cg24257309 | *AVP* | 20 | 3013423 | -5.61 | 2.90E-08 | 8.60E-05 | 55.0 | -2.6 | n/a | 0.09 |
| cg16353628 | *KCMF1* | 2 | 85093427 | -5.60 | 3.00E-08 | 8.70E-05 | 76.9 | -0.6 | n/a | 0.08 |
| cg04094640 | *NSMCE1* | 16 | 27144623 | -5.60 | 3.10E-08 | 8.80E-05 | 34.9 | -0.4 | n/a | 0.10 |
| cg15961007 | *MYCT1* | 6 | 153064972 | -5.59 | 3.10E-08 | 8.90E-05 | 76.1 | -2.8 | -0.35 | n/a |
| cg18619616 | *N/A* | 7 | 23301402 | -5.59 | 3.30E-08 | 9.10E-05 | 65.2 | 0.8 | -0.44 | 0.16 |
| cg23414595 | *ZMAT5* | 22 | 28459607 | -5.57 | 3.50E-08 | 9.80E-05 | 52.1 | -1.8 | -0.17 | n/a |
| cg16586538 | *PPP1R10* | 6 | 30677084 | -5.57 | 3.60E-08 | 9.80E-05 | 71.7 | -1.2 | -0.13 | n/a |
| cg10584478 | *BAHCC1* | 17 | 77037125 | -5.57 | 3.70E-08 | 1.01E-04 | 70.8 | n/a | -0.47 | 0.11 |
| cg23580000* | *ADCY7* | 16 | 48879657 | -5.55 | 4.00E-08 | 1.09E-04 | 66.3 | -0.8 | -0.20 | 0.11 |
| cg01044849 | *NCRNA00171* | 6 | 30110702 | -5.54 | 4.20E-08 | 1.13E-04 | 72.5 | -1.4 | -0.36 | n/a |
| cg01799671 | *CMIP* | 16 | 80118253 | -5.52 | 4.60E-08 | 1.24E-04 | 49.8 | -4.2 | n/a | 0.22 |
| cg02017450 | *N/A* | 10 | 10671676 | -5.52 | 4.80E-08 | 1.29E-04 | 61.0 | -7.1 | n/a | 0.23 |
| cg01409343 | *TMEM49* | 17 | 55270522 | -5.51 | 5.00E-08 | 1.32E-04 | 66.8 | n/a | n/a | n/a |
| cg24020157* | *RASGEF1A* | 10 | 43017527 | -5.51 | 5.10E-08 | 1.33E-04 | 50.9 | -2.3 | n/a | 0.18 |
| cg06311778 | *TENC1* | 12 | 51727849 | -5.50 | 5.40E-08 | 1.38E-04 | 21.5 | -0.6 | n/a | 0.11 |
| cg24453118 | *LRCH1* | 13 | 46127928 | -5.50 | 5.40E-08 | 1.38E-04 | 61.5 | -5.2 | n/a | n/a |
| cg14765414 | *MCF2L* | 13 | 112700336 | -5.49 | 5.70E-08 | 1.45E-04 | 41.1 | 0.2 | n/a | n/a |
| cg09091752 | *DNAJC17* | 15 | 38867650 | -5.48 | 5.70E-08 | 1.45E-04 | 64.2 | -0.6 | -0.09 | 0.07 |
| cg27106909 | *YPEL3* | 16 | 30014398 | -5.48 | 5.90E-08 | 1.47E-04 | 38.4 | -1.5 | 0.20 | n/a |
| cg11118235* | *GNAI2* | 3 | 50259014 | -5.48 | 6.00E-08 | 1.51E-04 | 35.4 | -1.8 | -0.13 | 0.06 |
| cg25197194 | *CCDC48* | 3 | 130241477 | -5.47 | 6.20E-08 | 1.52E-04 | 64.3 | -1.3 | 0.14 | 0.13 |
| cg02291556 | *PDZK1IP1* | 1 | 47428727 | -5.47 | 6.20E-08 | 1.53E-04 | 33.3 | -0.2 | -0.31 | 0.14 |
| cg08866634 | *RNF44* | 5 | 175894944 | -5.46 | 6.40E-08 | 1.57E-04 | 40.6 | -2.0 | -0.16 | 0.14 |
| cg13607248 | *DVL3* | 3 | 185357250 | -5.46 | 6.60E-08 | 1.60E-04 | 54.1 | -2.3 | n/a | 0.12 |
| cg10501360 | *ARHGAP22* | 10 | 49341671 | -5.46 | 6.70E-08 | 1.63E-04 | 82.2 | -1.7 | -0.08 | n/a |
| cg02217713 | *PRKAR1B* | 7 | 609681 | -5.46 | 6.70E-08 | 1.63E-04 | 57.0 | 0.2 | -0.43 | 0.13 |
| cg12054453 | *TMEM49* | 17 | 55270499 | -5.45 | 6.80E-08 | 1.63E-04 | 35.2 | n/a | 0.19 | 0.16 |
| cg03485667 | *ZNRF1* | 16 | 73700701 | -5.45 | 7.00E-08 | 1.66E-04 | 67.4 | -2.4 | -0.25 | 0.09 |
| cg20559943 | *N/A* | 4 | 186057750 | -5.45 | 7.10E-08 | 1.68E-04 | 28.7 | -0.4 | n/a | n/a |
| cg20820107 | *TNFRSF8* | 1 | 12115686 | -5.44 | 7.40E-08 | 1.75E-04 | 17.8 | n/a | -0.12 | n/a |
| cg10713589 | *N/A* | 4 | 120209400 | -5.44 | 7.50E-08 | 1.75E-04 | 56.9 | -3.5 | n/a | 0.22 |
| cg08783253 | *AOC2* | 17 | 38250091 | -5.43 | 7.60E-08 | 1.78E-04 | 71.6 | -1.5 | -0.33 | 0.14 |
| cg09229492 | *NKD1* | 16 | 49184079 | -5.43 | 7.70E-08 | 1.78E-04 | 71.0 | -0.4 | n/a | n/a |
| cg08631357 | *SLC6A7* | 5 | 149569403 | -5.43 | 7.70E-08 | 1.78E-04 | 35.8 | -0.8 | 0.11 | 0.08 |
| cg27165456 | *ELFN1* | 7 | 1749103 | -5.43 | 7.80E-08 | 1.78E-04 | 37.2 | -1.2 | -0.23 | 0.15 |
| cg13597417* | *LGALS9C* | 17 | 18321079 | -5.43 | 7.80E-08 | 1.78E-04 | 16.0 | -0.6 | n/a | 0.08 |
| cg20600850* | *CHD4* | 12 | 6582776 | -5.43 | 7.90E-08 | 1.78E-04 | 30.0 | -2.1 | n/a | n/a |
| cg19115490* | *N/A* | 6 | 37591903 | -5.42 | 8.10E-08 | 1.82E-04 | 30.7 | -1.3 | n/a | n/a |
| cg11882607 | *CAND2* | 3 | 12833926 | -5.41 | 8.60E-08 | 1.93E-04 | 65.8 | -1.7 | n/a | 0.09 |
| cg05581878 | *N/A* | 3 | 161369675 | -5.41 | 8.70E-08 | 1.95E-04 | 77.9 | -4.0 | n/a | n/a |
| cg25009965 | *ZBTB16* | 11 | 113536330 | -5.40 | 8.90E-08 | 1.97E-04 | 29.0 | -0.8 | n/a | 0.15 |
| cg12103569 | *SFRS8* | 12 | 130824513 | -5.39 | 9.40E-08 | 2.07E-04 | 40.8 | -0.5 | 0.49 | n/a |
| cg09915396* | *RAP1GAP2* | 17 | 2854645 | -5.39 | 9.70E-08 | 2.12E-04 | 39.6 | -1.2 | n/a | 0.16 |
| cg03930153 | *TBL1XR1* | 3 | 178351590 | -5.39 | 9.80E-08 | 2.13E-04 | 55.2 | -4.3 | n/a | 0.16 |
| cg17133774* | *CHD5* | 1 | 6121254 | -5.38 | 9.90E-08 | 2.15E-04 | 31.5 | -3.0 | n/a | 0.24 |
| Probes with positive association between gestational age and methylation in the ARIES cohort only | | | | | | | | | | |
| cg07738730* | *IGF2BP1* | 17 | 44432164 | 8.72 | 1.90E-17 | 9.40E-13 | 47.5 | 3.1 | -0.17 | -0.23 |
| cg18183624 | *IGF2BP1* | 17 | 44431903 | 6.67 | 5.10E-11 | 4.90E-07 | 44.3 | 3.5 | -0.22 | -0.25 |
| cg14748380* | *FAM163A* | 1 | 177980411 | 6.61 | 7.60E-11 | 7.00E-07 | 38.7 | 3.2 | n/a | -0.18 |
| cg10927968 | *N/A* | 11 | 1763909 | 6.32 | 4.50E-10 | 2.90E-06 | 61.2 | 1.4 | n/a | -0.20 |
| cg12001120* | *WNT10A* | 2 | 219459586 | 6.27 | 6.10E-10 | 3.80E-06 | 65.5 | 2.0 | n/a | -0.14 |
| cg25592206 | *CDKN2C* | 1 | 51211936 | 6.17 | 1.10E-09 | 6.20E-06 | 69.6 | 1.0 | -0.13 | -0.12 |
| cg05460329* | *CLDN5* | 22 | 17892903 | 6.16 | 1.20E-09 | 6.50E-06 | 76.2 | 1.3 | n/a | -0.11 |
| cg06704969 | *C3orf21* | 3 | 196278388 | 6.11 | 1.60E-09 | 7.80E-06 | 74.8 | 0.7 | n/a | -0.16 |
| cg16426670* | *EBF4* | 20 | 2623996 | 6.11 | 1.60E-09 | 7.80E-06 | 50.1 | 2.8 | -0.11 | -0.14 |
| cg00499707 | *N/A* | 20 | 34143079 | 6.05 | 2.30E-09 | 1.10E-05 | 76.4 | 0.8 | -0.08 | -0.07 |
| cg05017199 | *TMEM176B* | 7 | 150127998 | 5.97 | 3.80E-09 | 1.60E-05 | 49.1 | 1.9 | -0.18 | -0.09 |
| cg13518079 | *EBF4* | 20 | 2623072 | 5.97 | 3.80E-09 | 1.60E-05 | 13.7 | 3.6 | -0.16 | n/a |
| cg24460126 | *FAM163A* | 1 | 177980451 | 5.89 | 5.80E-09 | 2.40E-05 | 60.5 | 2.8 | n/a | -0.15 |
| cg27184903* | *APBA2* | 15 | 27073019 | 5.89 | 5.90E-09 | 2.40E-05 | 47.4 | 1.7 | n/a | -0.13 |
| cg08654091 | *N/A* | 1 | 225082041 | 5.89 | 6.00E-09 | 2.40E-05 | 42.4 | 1.4 | n/a | -0.18 |
| cg26077811 | *USP2* | 11 | 118737473 | 5.87 | 6.70E-09 | 2.60E-05 | 51.1 | 1.8 | n/a | -0.17 |
| cg04463638 | *CLDN5* | 22 | 17892712 | 5.82 | 8.90E-09 | 3.40E-05 | 52.7 | 3.6 | n/a | -0.14 |
| cg05857996 | *EBF4* | 20 | 2623418 | 5.81 | 9.30E-09 | 3.50E-05 | 26.0 | 6.7 | n/a | -0.21 |
| cg16773741 | *CLDN5* | 22 | 17893017 | 5.77 | 1.20E-08 | 4.00E-05 | 72.4 | 1.8 | n/a | -0.11 |
| cg00889363 | *PARD3B* | 2 | 205964086 | 5.74 | 1.40E-08 | 4.70E-05 | 44.3 | 1.7 | -0.15 | -0.14 |
| cg16107105* | *KCNH2* | 7 | 150277637 | 5.72 | 1.50E-08 | 5.00E-05 | 74.6 | 1.2 | n/a | -0.08 |
| cg19434937 | *LPCAT3* | 12 | 6974445 | 5.72 | 1.60E-08 | 5.00E-05 | 72.5 | n/a | 0.13 | n/a |
| cg06238004* | *N/A* | 17 | 39385253 | 5.66 | 2.20E-08 | 6.80E-05 | 57.1 | 4.0 | n/a | -0.13 |
| cg11934832 | *N/A* | 18 | 43937839 | 5.65 | 2.30E-08 | 6.80E-05 | 72.7 | 1.0 | 0.10 | -0.09 |
| cg20722590 | *MMP15* | 16 | 56618974 | 5.60 | 3.00E-08 | 8.60E-05 | 59.2 | 1.4 | -0.13 | n/a |
| cg25096368 | *N/A* | 12 | 52436883 | 5.60 | 3.00E-08 | 8.70E-05 | 59.2 | 1.1 | -0.13 | n/a |
| cg18217136 | *BLCAP* | 20 | 35591065 | 5.58 | 3.40E-08 | 9.50E-05 | 69.0 | 0.2 | n/a | n/a |
| cg19851574 | *RPS6KA2* | 6 | 167098223 | 5.56 | 3.80E-08 | 1.03E-04 | 65.6 | n/a | 0.24 | -0.18 |
| cg19759478 | *C1orf183* | 1 | 112093399 | 5.54 | 4.30E-08 | 1.14E-04 | 56.5 | 1.6 | 0.10 | -0.10 |
| cg21363811 | *FAM163A* | 1 | 177980573 | 5.50 | 5.20E-08 | 1.35E-04 | 55.2 | 2.5 | n/a | -0.12 |
| cg03684807 | *N/A* | 22 | 44836048 | 5.50 | 5.30E-08 | 1.37E-04 | 27.9 | 5.4 | -0.36 | -0.10 |
| cg14427590 | *N/A* | 17 | 58048821 | 5.48 | 5.80E-08 | 1.46E-04 | 60.7 | 0.4 | -0.07 | -0.08 |
| cg15908709* | *LOC404266* | 17 | 44031214 | 5.45 | 6.80E-08 | 1.64E-04 | 34.8 | 2.8 | -0.49 | -0.15 |
| cg13871826 | *CYR61;DDAH1* | 1 | 85817935 | 5.42 | 8.00E-08 | 1.80E-04 | 62.3 | 1.9 | n/a | -0.07 |
| cg18147098 | *N/A* | 1 | 210755539 | 5.40 | 9.20E-08 | 2.03E-04 | 44.5 | 4.4 | n/a | -0.14 |

*also identified in case control study of pre-term birth by Cruickshank et al (2013)
